# An altered microbiome in urban coyotes mediates relationships between anthropogenic diet and poor health

**DOI:** 10.1038/s41598-020-78891-1

**Published:** 2020-12-17

**Authors:** Scott Sugden, Dana Sanderson, Kyra Ford, Lisa Y. Stein, Colleen Cassady St. Clair

**Affiliations:** 1 Department of Biological Sciences, University of Alberta, CW405 Biological Sciences Building, Edmonton, AB T6G 2E9 Canada; 2 Department of Biological Sciences, MacEwan University, Edmonton, AB Canada

**Keywords:** Microbial ecology, Urban ecology

## Abstract

Generalist species able to exploit anthropogenic food sources are becoming increasingly common in urban environments. Coyotes (*Canis latrans*) are one such urban generalist that now resides in cities across North America, where diseased or unhealthy coyotes are frequently reported in cases of human-wildlife conflict. Coyote health and fitness may be related to habitat use and diet via the gut microbiome, which has far-reaching effects on animal nutrition and physiology. In this study, we used stomach contents, stable isotope analysis, 16S rRNA gene amplicon sequencing, and measures of body condition to identify relationships among habitat use, diet, fecal microbiome composition, and health in urban and rural coyotes. Three distinct relationships emerged: (1) Urban coyotes consumed more anthropogenic food, which was associated with increased microbiome diversity, higher abundances of *Streptococcus* and *Enterococcus*, and poorer average body condition. (2) Conversely, rural coyotes harbored microbiomes rich in Fusobacteria, *Sutterella,* and *Anaerobiospirillum*, which were associated with protein-rich diets and improved body condition. (3) Diets rich in anthropogenic food were associated with increased abundances of *Erysipelotrichiaceae*, *Lachnospiraceae*, and *Coriobacteriaceae*, which correlated with larger spleens in urban coyotes. Urban coyotes also had an increased prevalence of the zoonotic parasite *Echinococcus multilocularis*, but there were no detectable connections between parasite infection and microbiome composition. Our results demonstrate how the consumption of carbohydrate-rich anthropogenic food by urban coyotes alters the microbiome to negatively affect body condition, with potential relationships to parasite susceptibility and conflict-prone behavior.

## Introduction

Urbanization is causing dramatic changes to terrestrial ecosystems. For many species, the selective pressures created by the expanding urban landscape^1,2^ lead to local extirpations and consequent decreases in local biodiversity^3^, but several generalist species can thrive in urban environments^4–6^. The success of these urban generalists is largely enabled by behavioral adaptations, foremost of which is broadening their diet to exploit abundant but often variable sources of anthropogenic food^4^. Urban habitat use has been loosely associated with physical costs^7^, including unhealthy weight gain^8^ and increased cholesterol and corticosterone levels^9,10^. However, the direct physiological consequences of eating anthropogenic food remain less well understood, despite likely downstream effects on human-wildlife interactions including dependency, conflict-prone behavior, and the spread of zoonotic diseases^11^.

The gut microbiome may play a pivotal role linking the consumption of anthropogenic food by urban wildlife to changes in their physiology and ecology because it is necessarily altered by changes in diet and has far-reaching effects on nutrient assimilation, immune system function, and overall fitness^12,13^. Healthy, diverse gut microbiomes facilitate host digestion and nutrition^14^ and can act as a barrier to infection^15^. These effects have direct implications for host health and behavior. For example, variations in the animal gut microbiota have been linked to parasite susceptibility^16^ and body condition^17^, and an altered gut microbiota has been associated with aggression in neglected (and presumably malnourished) canines^18^. In some cases, specific bacterial taxa, such as *Bifidobacterium* and *Lactobacillus* in humans, have been identified for their beneficial effects on host health^19^.

Microbiome research continues to demonstrate the importance of the microbiome across diverse animal hosts in the context of environmental perturbation. Urban-induced alterations in the microbiome have been observed in wild birds^20–23^ and linked to physiological stress in squirrels^24^. Disturbances related to urbanization have been shown to affect microbiome diversity and composition in several animal species^25–27^. Some authors have suggested that these anthropogenic threats to host-associated microbial communities have important implications for wildlife management practices^28^. However, for information about the effects of microbiome alterations in urban wildlife to be meaningful to managers, it must also be accompanied by an understanding of the “healthy” microbiome in each species. Moreover, most current descriptions of the animal microbiome draw primarily from captive or model organisms, but captivity itself affects the microbiome^29,30^ and, due to natural physiological differences among animal taxa, results from one species cannot reliably be extrapolated to another^31^. It is therefore important to study directly how the complex process of urban adaptation affects the microbiome of different urban-adapted species.

In North America, coyotes (*Canis latrans*) are becoming a common resident of most major cities, which has coincided with increased reports of human-coyote conflict^32^. Their success in urban environments stems from their generalist and flexible diet^32^ and behavioral plasticity^33^. Although coyotes mainly consume insects, rodents, and young or diseased ungulates^34^, they may also consume fruit and anthropogenic food^32,35^. They are also a definitive host for the helminth parasite *Echinococcus multilocularis*, which is expanding its range in North America and can cause a rare but severe zoonosis in humans^36^. Previous work in our lab has shown that urban and conflict-prone coyotes eat less protein^35^, that consumption of anthropogenic food in urban coyotes is correlated with higher prevalence of parasites^37,38^, and that urban coyotes have an unusually high prevalence of *E. multilocularis*^39^. Knowledge of which microbial signatures are associated with diet, body condition, or parasite infection in coyotes would not only provide novel insights into host-microbiome relationships in wild canids but also have direct management implications for evaluating or monitoring host fitness, especially in the contexts of potential conflict or the spread of canid-borne zoonoses.

In this study, we tested the hypothesis that the consumption of anthropogenic food by urban coyotes causes a quantifiable shift in microbiome composition, with consequent declines in physiological condition. Using a sample of coyotes from urban and rural areas near Edmonton, Alberta, we identified specific relationships among diet, fecal bacterial communities, and host health, with an emphasis on generating a foundational understanding of which microbiome features most strongly connect diet to health in a wild canid. Our results support past evidence that urban coyotes eat a broader diet of lower quality, and additionally show that this is associated with poorer average body condition, increased immune stress, and a higher prevalence of a zoonotic parasite. Moreover, our results implicate specific bacterial taxa that characterize this shift in urban coyotes and provide an important basis for understanding how diet, microbiome composition, and health may interact to shape human-wildlife relationships in urban areas.

## Results

We obtained 30 road-killed or lethally managed coyote carcasses from Edmonton, Alberta (“urban”) and 65 coyotes that were collected or lethally managed by local fur trappers working in the surrounding area (“rural”) (Table S1). For each coyote, we measured body condition and age, and we evaluated short-term diet (i.e., past 6–8 h) using stomach contents and long-term diet (i.e., past 6–8 months) using stable isotopes. We additionally used 16S rRNA gene amplicon sequencing to characterize fecal bacterial communities and PCR to test for *E. multilocularis* infection. Because many physiological measures were correlated with each other, we performed dimension reduction to generate a single index of physical condition. Spleen mass, which serves as an indicator of immune function in mammals^40^, was corrected for body mass and retained separately from the composite physical condition index. Our coyotes exhibited expected sex differences in size, with males being larger than females, but there were no sex- or age-based differences in diet, measured as either stomach contents or stable isotope values (Table 1; Table S2).

**Table 1:** ANOVA results evaluating the effects of sex, age, and capture location on measures of diet, body condition, and microbiome alpha diversity.

	Mean Value		Sex			Age			Location		
	Urban	Rural	F	df	p	F	df	p	F	df	P
Mass (kg)	10.30	10.85	23.48	1	**< 0.001**	48.90	1	**< 0.001**	0.34	1	0.725
Body size (cm)	84.87	87.31	8.08	1	**0.006**	31.94	1	**< 0.001**	1.44	1	0.233
Girth (cm)	45.73	46.87	11.29	1	**< 0.001**	25.70	1	**< 0.001**	0.17	1	0.684
Age (years)	1.82	2.64	5.70	1	**0.019**	–	–	–	3.18	1	0.078
Spleen size (g/kg)	2.34	1.70	1.20	1	0.276	2.51	1	0.117	28.01	1	**< 0.001**
KFI	0.32	0.57	2.06	1	0.150	1.68	1	0.198	15.14	1	**< 0.001**
Health index	− 0.02	0.01	13.27	1	**< 0.001**	40.93	1	**< 0.001**	0.01	1	0.316
δ^13^C (‰)	− 21.48	− 22.90	0.01	1	0.926	1.38	1	0.243	54.11	1	**< 0.001**
δ^15^N (‰)	8.69	8.96	1.53	1	0.220	1.98	1	0.163	2.24	1	0.138
Vol. anthro food (ml)	34.42	24.61	0.07	1	0.793	3.21	1	0.076	0.24	1	0.627
Vol. prey (ml)	59.97	211.58	1.06	1	0.306	1.21	1	0.274	5.75	1	**0.018**
ASV richness	333.40	294.28	0.12	1	0.738	0.90	1	0.344	1.93	1	0.060
Shannon index	3.11	2.68	0.48	1	0.492	0.17	1	0.678	5.71	1	**0.019**
Faith's PD	44.27	31.34	0.37	1	0.546	1.25	1	0.270	38.68	1	**< 0.001**
NTI	6.47	4.40	1.89	1	0.173	1.37	1	0.245	33.11	1	**< 0.001**

All variables were tested using a linear regression, with the exception of ASV richness, which was tested using a negative binomial regression. Stomach contents were log-transformed prior to testing to meet the assumptions of a normal distribution.

*KFI* kidney fat index, *ASV* amplicon sequence variant, *PD* phylogenetic diversity, *NTI* nearest taxon index.

### Coyote diet and health

**Figure 1:**
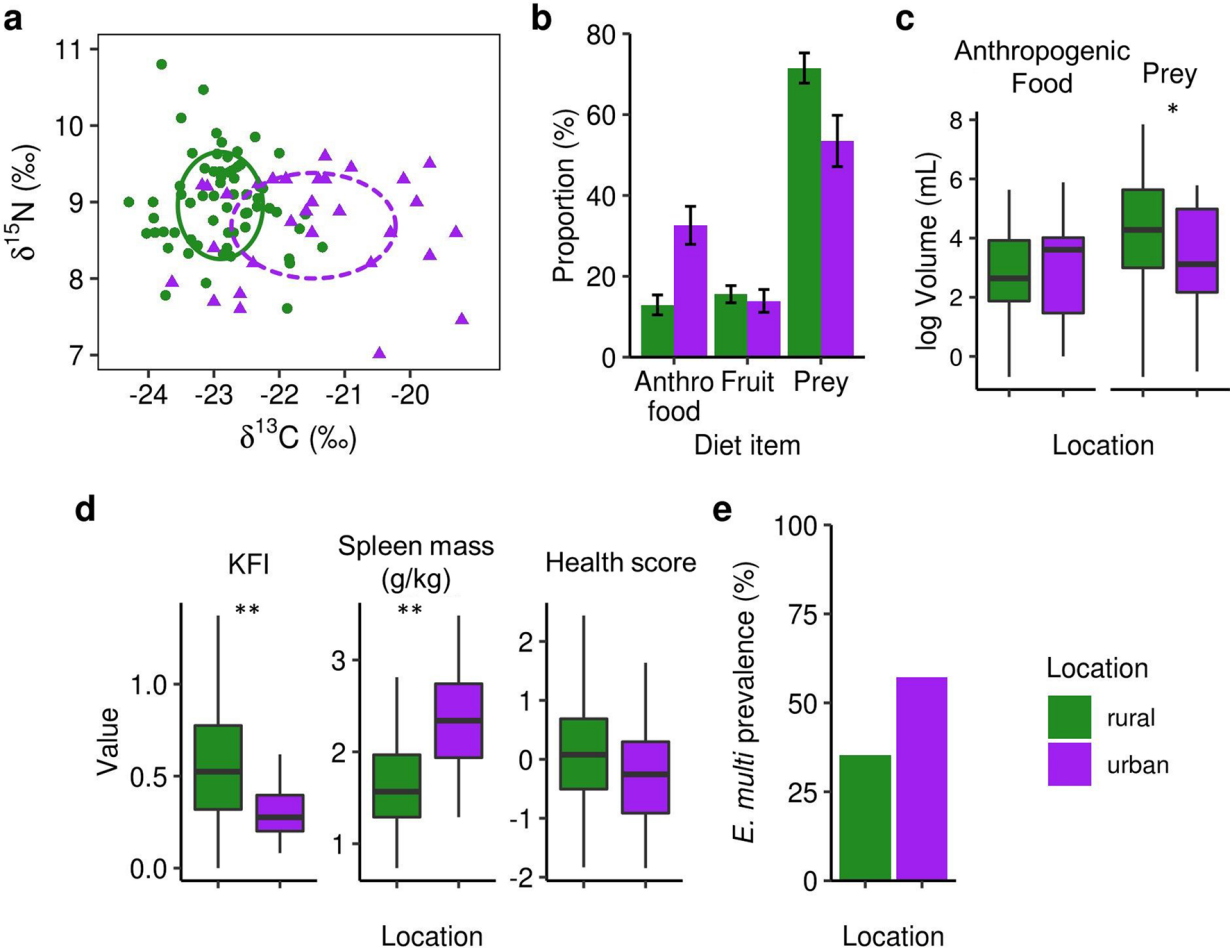
Diet and body condition in urban and rural coyotes. (**a**) Stable isotope isoscape based on δ^13^C and δ^15^N values, showing that urban coyotes have higher δ^13^C and lower δ^15^N signatures. (**b**) Results from a three- source stable isotope mixing model predicting the proportions of prey, fruit, and anthropogenic food in coyote diets. (**c**) Mean volumes of stomach contents in urban and rural coyotes. (**d**) Urban coyotes have less kidney fat and larger spleens than rural coyotes and perform slightly worse on a composite index of physical condition. (**e**) Prevalence of *E. multilocularis* in urban and rural coyotes. In all figures, significant differences are indicated by asterisks (* for p < 0.05; ** for p < 0.01).

Urban coyotes consumed more anthropogenic food (e.g., compost and fast food waste) and less prey (e.g., small herbivores and ungulates) than rural coyotes. To assess habitual diet, we used stable isotope values (δ^13^C and δ^15^N) measured from claw samples. Stable isotopes can capture trends in anthropogenic food consumption because corn, which is ubiquitous in processed foods and livestock feed, has a distinctively high δ^13^C signature^41^. Higher δ^15^N signatures indicate greater protein consumption^41^. In our study, urban coyotes had a significantly higher mean δ^13^C signature than rural coyotes, as well as a lower mean δ^15^N signature, when controlling for sex and age (Fig. 1a; Table 1). Stable isotope mixing models estimated that urban coyotes consumed 2.5 times more anthropogenic food and 25% less prey than rural coyotes (Fig. 1b). We used stomach contents as a measure of short-term diet and found that urban coyotes were less likely to have natural prey in their stomach (70% vs. 84.6%), though this difference was only marginally significant (χ^2^ = 3.25, df = 1, p = 0.071). If natural prey was present, it was present at significantly lower volumes (Fig. 1c; Table 1). There were no significant differences in the presence or volume of anthropogenic food between urban and rural coyote stomachs (Table 1; Table S3).

In addition to assimilating a more anthropogenic diet, urban coyotes exhibited poorer body condition than rural coyotes. Urban coyotes had half as much kidney fat, which we measured as the kidney fat index (KFI), an indicator of body fat reserves^42^ (Fig. 1d; Table 1). After controlling for body mass, urban coyotes also had 37% larger spleens (Fig. 1d; Table 1), suggesting they may be experiencing more challenges to their immune system^40^. We lastly found that urban coyotes were 50% more likely to carry the intestinal helminth *E. multilocularis* (Fig. 1e; 53% vs. 35% prevalence), and this increase was marginally significant (χ^2^ = 3.80, df = 1, p = 0.051). *E. multilocularis* was also more common in younger coyotes (Fig. S1). These changes in kidney fat, spleen mass, and infection prevalence were not confounded by any differences in body mass, size, or age (Table 1).

### Fecal microbiome composition

**Figure 2:**
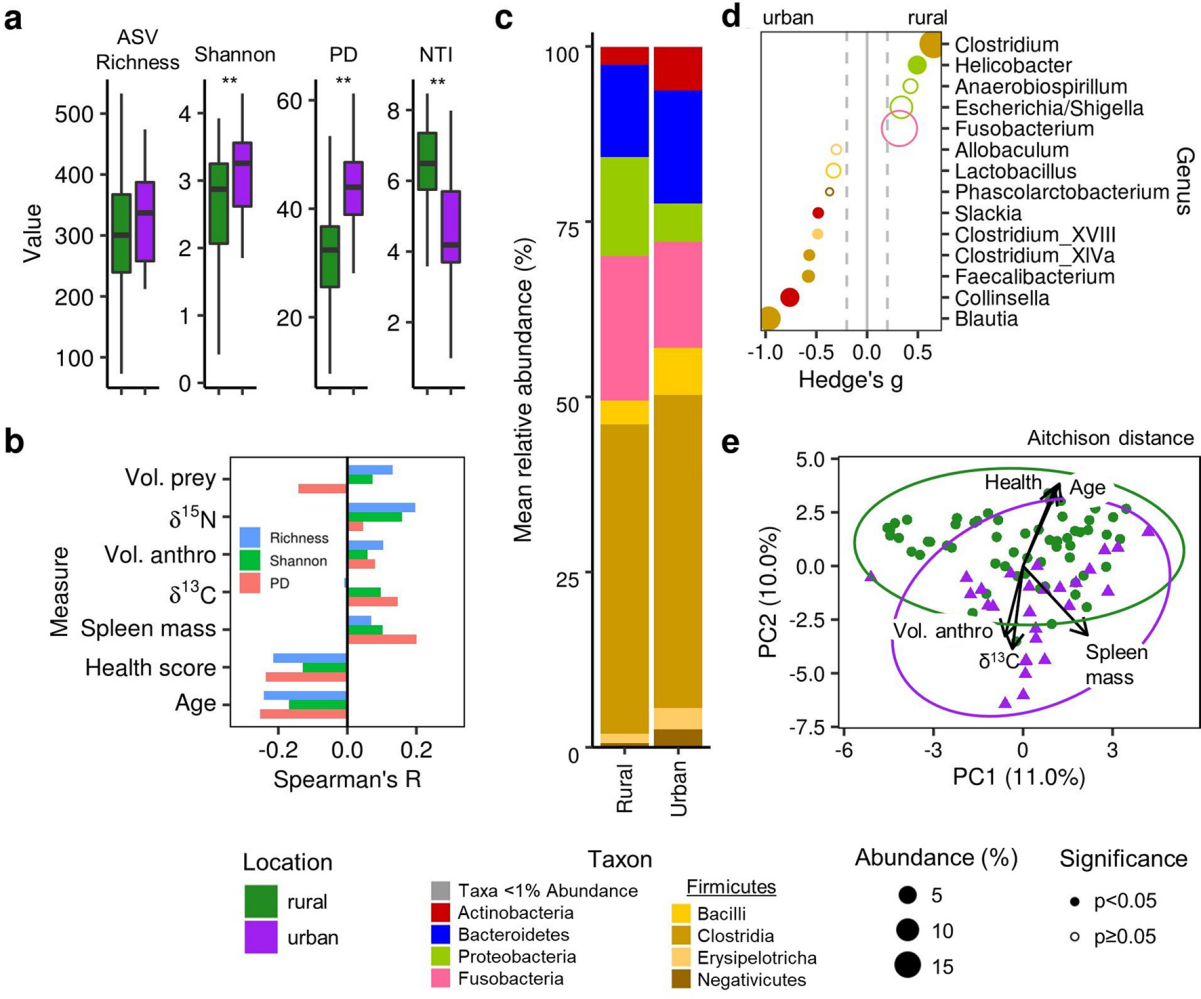
Coyote fecal microbiome alpha-diversity, composition, and structure. (**a**) Differences in measures of ASV richness, Shannon diversity, Faith’s phylogenetic diversity (PD), and the nearest taxon index (NTI) between the fecal microbiome of urban and rural coyotes. Significant differences (p < 0.01) are indicated by asterisks. (**b**) Spearman correlation coefficients between richness, diversity, and PD and measures of coyote diet and body condition. (**c**) Average relative abundance of each taxon in urban and rural coyotes. Taxa with a mean relative abundance of < 1% are categorized as ‘other.’ Firmicutes (yellow shades) are separated to the class level; all other taxa appear at the phylum level. (**d**) Differentially abundant bacterial families between urban and rural coyotes, ranked by the effect-size difference between groups measured using Hedge’s *g*. Circle size indicates taxon relative abundance, and solid circles indicate significantly differentially abundant taxa (Benjamini–Hochberg adjusted p < 0.05). (**e**) Principal components analysis using the Aitchison distance, which is based on centered log-ratio transformed ASV abundances. Vectors indicate significant (p < 0.05) relationships with diet and health measures.

We used 16S rRNA gene amplicon sequencing to investigate how the fecal microbiome may mediate the relationships between an anthropogenic diet and poor health in urban coyotes, and, equivalently, between protein-rich diets and good health in rural coyotes. Shannon diversity and phylogenetic diversity (PD) were significantly higher in urban coyotes (Fig. 2a, Table 1). ASV richness was also moderately higher in urban coyotes and, after controlling for location, in coyotes infected with *E. multilocularis* (Fig. S2), though these increases were only marginally significant (Table 1, Fig. S2). Generalized linear models (GLMs) confirmed that the best predictors of richness and Shannon diversity were coyote location and infection status (Fig. S3). No alpha diversity metrics were significantly associated with any measure of diet, but both richness and PD were moderately lower in healthier, older coyotes and in coyotes with empty stomachs (Fig. 2b; Figs. S3, S4). In addition, the nearest taxon index (NTI) was significantly lower in urban coyotes (Fig. 2a; Table 1). NTI can be used to assess phylogenetic clustering, or whether closely related species co-occur in an ecosystem; lower NTI values indicate that closely related species are less likely to co-occur, which suggests there may be more competitive interactions among taxa^43^. Aside from the effects of location, NTI was best predicted by the composite physical condition index and showed no relationship with diet (Fig. S3).

The differences in microbiome diversity between urban and rural samples were reflected by altered abundances of several bacterial taxa (Fig. 2c). *Blautia*, *Collinsella,* and *Erysipelotrichaceae* spp. were significantly more abundant in urban samples, whereas *Clostridium* was significantly more abundant in rural samples (Fig. 2d). No taxa at any taxonomic level were significantly differentially abundant based on sex, age, or *E. multilocularis* infection (Fig. S5). Random forest models were 86% effective at discriminating samples by location, with almost perfect classification of rural samples (96.7%) compared to urban (64.3%). ASVs from *Lactobacillus* and *Fusobacterium* had the most discriminatory power and were more abundant in urban and rural samples, respectively (Fig. S6). Urban samples that were misclassified as rural based on their microbiome composition had lower alpha diversity measures and smaller spleens than those that were classified correctly (Fig. S7).

Urban and rural coyote microbiomes also significantly differed in overall community composition, assessed based on an Aitchison distance-based PERMANOVA controlling for age and sex (F = 2.83, R^2^ = 0.032, df = 1, p = 0.001; Fig. 2e; Table S4). These location-specific differences were consistent when using alternative distance metrics that account for either taxon presence or abundance and were magnified when evaluated using the UniFrac distance metrics, which account for phylogenetic relatedness among taxa (Fig. S8; Table S4). Health, age, spleen size, and the consumption of anthropogenic food also influenced microbiome community structure, mirroring the differences in these measures between urban and rural coyotes (Fig. 2e), but sex and *E. multilocularis* infection did not (Fig. S9).

### Taxa associated with health

**Figure 3:**
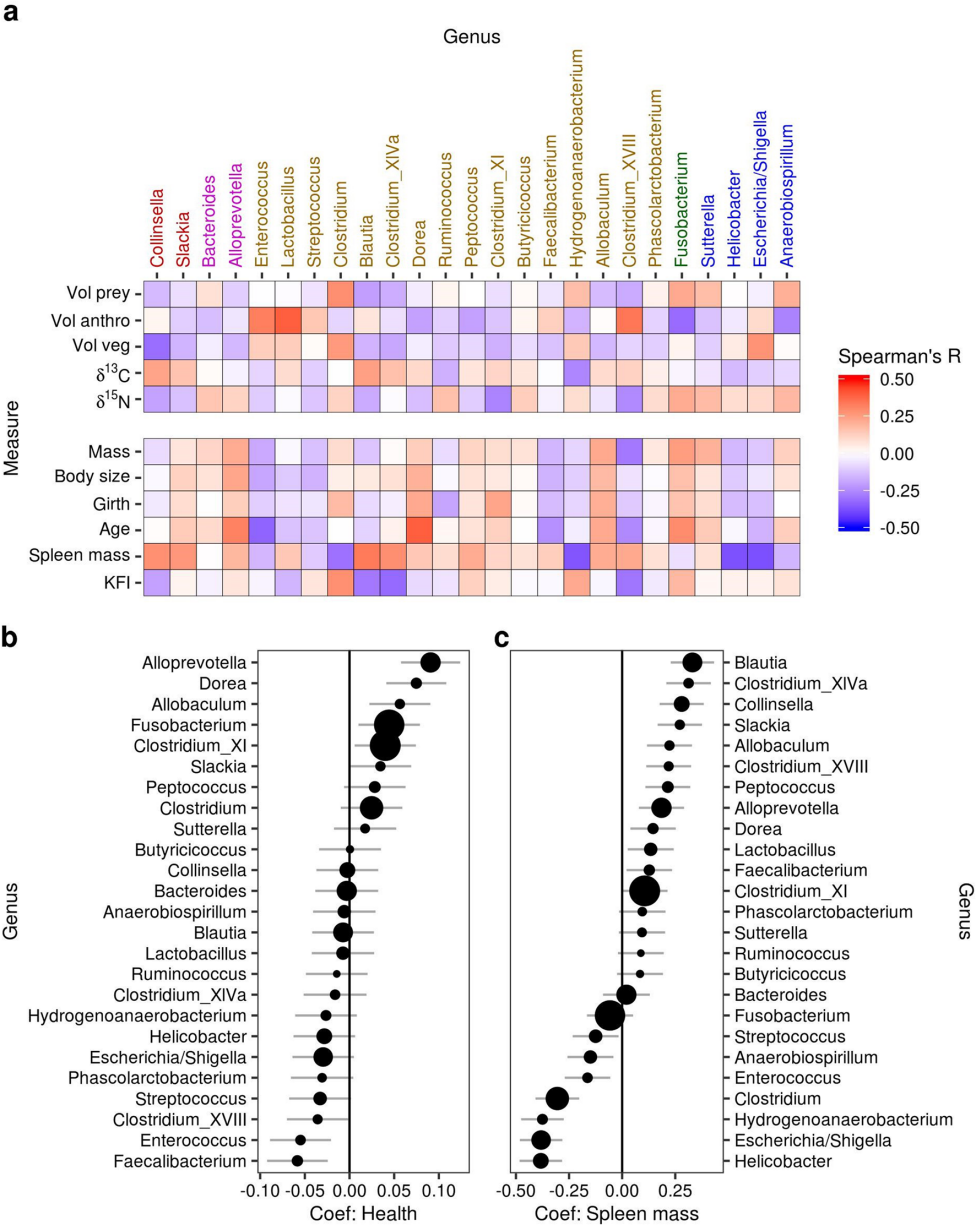
Univariate correlations between taxon abundances, diet, and body condition. (**a**) Heat map indicating Spearman’s correlation coefficient between centered log-ratio transformed taxon abundances and coyote metadata. (**b**,**c**) Results from rank-based regression models that used taxon abundances to predict (**b**) physical condition and (**c**) spleen mass, while controlling for the effects of sex. Error bars indicate standard error.

To examine how individual taxa responded to variation in coyote diet and body condition, we tested for correlations between taxon abundances and coyote metadata using both univariate and multivariate approaches. Based on Spearman’s correlation and rank-based regression models, *Alloprevotella, Dorea,* and *Allobaculum* were the best predictors of higher body condition, followed by the dominant genera *Fusobacterium* and *Clostridium XI* (Fig. 3a,b). These taxa were also positively correlated with age and both short- and long-term consumption of prey (Fig. 3a). *Faecalibaterium, Enterococcus, Helicobacter, Clostridium* cluster XVIII, and *Streptococcus* were the best predictors of poor body condition (Fig. 3a,b). Univariate approaches also showed that *Blautia, Clostridium XIVa, Collinsella*, and *Slackia* were associated with larger spleens, younger coyotes, and the consumption of anthropogenic food (Fig. 3a,c).

**Figure 4:**
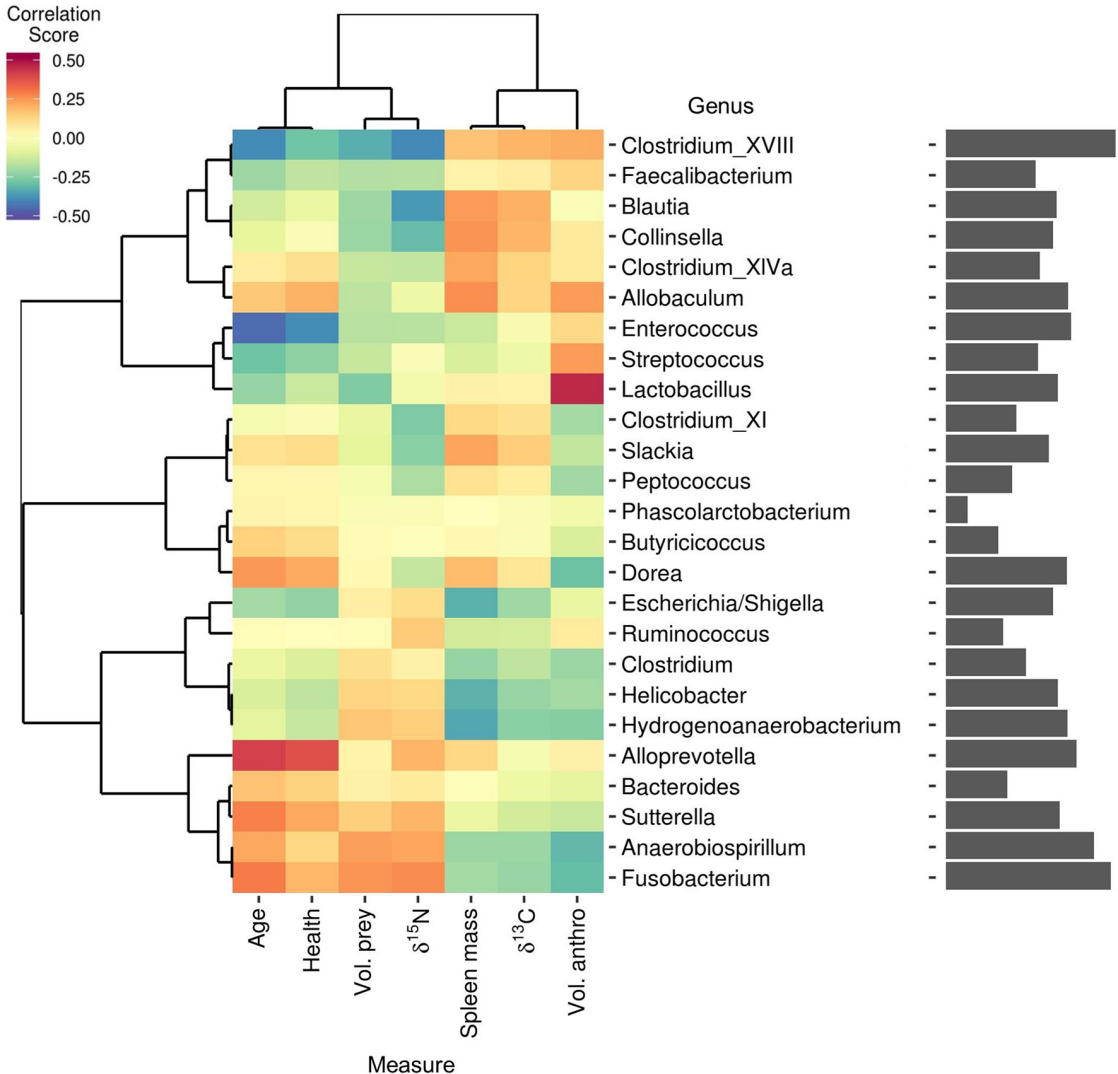
Multivariate associations among taxon abundances, diet, and body condition. Clustered image map relating the 25 most prevalent and abundant bacterial genera to coyote health and diet information. Colors indicate similarity measures obtained from regularized canonical correlation analysis (rCCA). Dendrograms were obtained by correlation distance-based hierarchical clustering of the similarity measures. Bars on the right indicate the relative importance of each genus in the network, calculated as the sum of the absolute values of the similarity measures.

In a multivariate approach, we used clustering associations from a regularized canonical correlation analysis (rCCA) to identify taxa that responded to measures of diet and health (Fig. 4). Explanatory variables separated into two clusters: (1) age and physical condition, which grouped with measures of protein consumption separately from measures of (2) anthropogenic food consumption and spleen mass (Fig. 4). There was a strong network of positive associations connecting *Fusobacterium, Anaerobiospirillum*, *Sutterella*, and *Bacteroides* to older age, the consumption of prey, and higher body condition. A similar network linked *Streptococcus, Enterococcus*, and *Lactobacillus* to younger coyotes, the volume of anthropogenic food in the stomach, and poor body condition (Fig. 4). A third grouping connected several genera of *Erysipelotrichaceae, Lachnospiraceae,* and *Coriobacteriaceae* to spleen mass, volume of anthropogenic food in the stomach, and δ^13^C, with variable effects on health (Fig. 4). These networks were validated using GLMs predicting the abundance of these taxa based on available explanatory variables (Figs. S10–S12).

**Figure 5:**
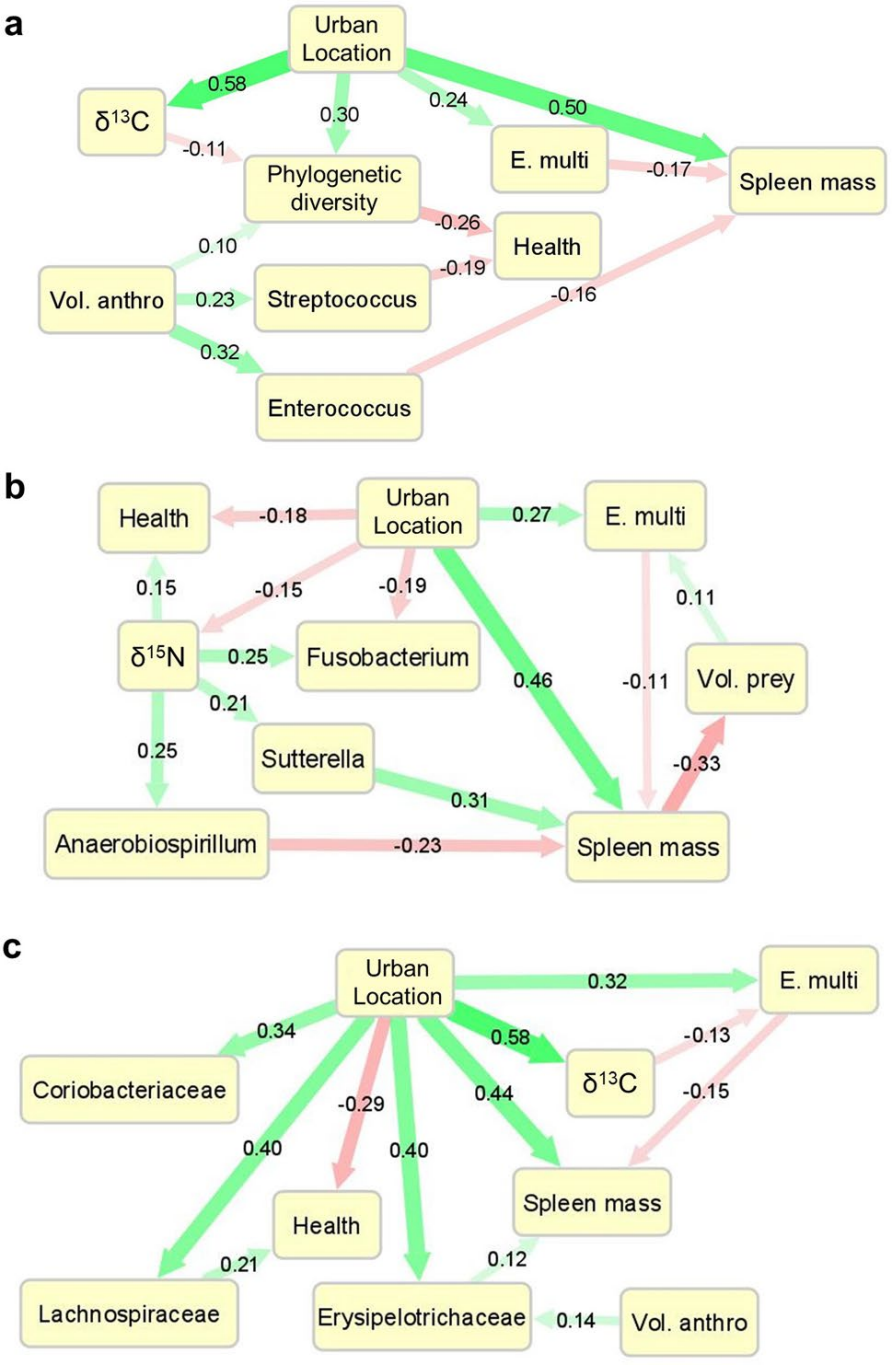
Structural equation models of factors associated with coyote health. Structural equation models were used to evaluate relationships among location, diet, microbiome composition, and health for microbiome features implicated in earlier analyses. The first model (**a**) focuses on the effects of anthropogenic food consumption; the second (**b**) focuses on the effects of protein consumption; and the third model (**c**) tests the taxa that were most strongly correlated with spleen mass. Model coefficients are standardized within each model.

We lastly used structural equation models (SEM) to model causal relationships among dietary measures and taxon abundances in the context of our questions about coyote location and health. In general, the best-fitting structural equation models supported relationships where urban coyotes consumed more anthropogenic food, which had indirect negative effects on health via increased phylogenetic diversity and increased abundances of *Enterococcus* and *Streptococcus* (Fig. 5a; Table S5). The SEM framework also supported paths where health in rural coyotes was connected to protein-rich diets; in these models, *Fusobacterium, Anaerobiospirillum*, and *Sutterella* served as indicators of a protein-rich diet but did not directly predict health (Fig. 5b; Table S5). A third model supported connections between anthropogenic food consumption, *Erysipelotrichaceae* spp., and spleen mass, with lesser effects of *Coriobacteriaceae* spp. and *Lachnospiraceae* spp. (Fig. 5c; Table S5). Notably, the model based on dietary protein supported a path in which the volume of prey in the stomach responded negatively to spleen mass, suggesting that immune function may affect coyote foraging success. In addition, the long-term dietary measure δ^15^N was the stronger predictor of downstream effects in protein-based models, whereas the short-term measure, volume of anthropogenic food in the stomach, was the stronger predictor in anthropogenic food-based models. All models predicted *E. multilocularis* infection as a function of location only, with no effect of diet or microbiome.

## Discussion

Generalist species able to survive on anthropogenic food are becoming increasingly common in urban environments, with important implications for human-wildlife interaction and conflict. Understanding the process of urban adaptation in these species requires understanding both how the gut microbiome responds to urban-associated changes in diet and how the microbiome relates to host fitness. In this study, urban coyotes consumed more anthropogenic food, had less assimilated kidney fat, displayed more signs of immune system stress, and were more likely to be infected with the zoonotic parasite *E. multilocularis*. These changes in diet and physiology were associated with distinct changes in fecal bacterial community composition. First, despite considerable evidence from humans and laboratory animals that gut microbiome diversity increases with age and health^44^, we found that the microbiomes of younger, less healthy urban coyotes were more diverse. In addition, we identified several bacterial taxa involved in the connection between diet and health, including some taxa known to be affected by diet in domestic dogs^45^. Specifically, *Streptococcus* and *Enterococcus* linked anthropogenic food consumption and poor health, whereas Fusobacteria, *Anaerobiospirillum,* and *Sutterella* were related to a protein-rich diet and improved body condition. Many of these microbial connections between diet and condition were preserved outside of the context of location and could therefore serve as non-invasive indicators of coyote health.

The relationships we observed linking the consumption of anthropogenic food by urban coyotes to increased microbiome diversity and increased abundances of *Streptococcus* and *Enterococcus* presumably stem from the altered macronutrient composition of an anthropogenic diet relative to conventional prey. Within a host species, much of the gut microbial alpha diversity is attributable to the range of microbial enzymatic capacities needed to degrade dietary nutrients^46^. This is why diets rich in plants and fibers lead to increased microbial diversity^47,48^. Anthropogenic foods contain a wider variety of macronutrients than prey^46^, particularly in the form of complex polysaccharides, and would therefore promote the higher phylogenetic diversity in the urban coyote gut that we observed here. These results disagree with previous studies in passerines that showed decreased microbiome diversity in urban birds^20,23^, but agree with another^22^, suggesting that how microbial diversity responds to urban habitat use may differ depending on host species, dietary components, or other aspects of an adaptation strategy.

The presumed increase in dietary carbohydrates associated with an anthropogenic diet would also contribute to the abundances of the lactic acid bacteria (LAB) *Streptococcus* and *Enterococcus*, with potential implications for overall condition. LAB are largely considered an important component of a healthy microbiome for omnivores and herbivores due to their role in digesting dietary carbohydrates^19^ and have been used as probiotics for many livestock species^49^. However, carnivore diets contain few carbohydrates^12^, and the increased abundance of LAB, as with the increase in diversity, may be a direct consequence of increased carbohydrate consumption. Despite these microbial responses to an altered diet, the digestive physiology of carnivores is not adapted to handle large amounts of carbohydrates, which presumably limits the amount of nutrition that can be effectively acquired from anthropogenic food and thus contributes to poor health^50^. Although this effect would be reflected, but not mediated, by the microbiome, select groups of *Streptococcaceae* and *Enteroccaceae* have been directly connected to negative health effects in humans^51^, and higher abundances of *Streptococcus* contribute to a dysbiosis index in dogs^52^. Taxon abundances were also better than diet at predicting condition in structural equation models, and it is therefore possible that some of these taxa may directly influence, rather than indicate, poor condition.

**Figure 6:**
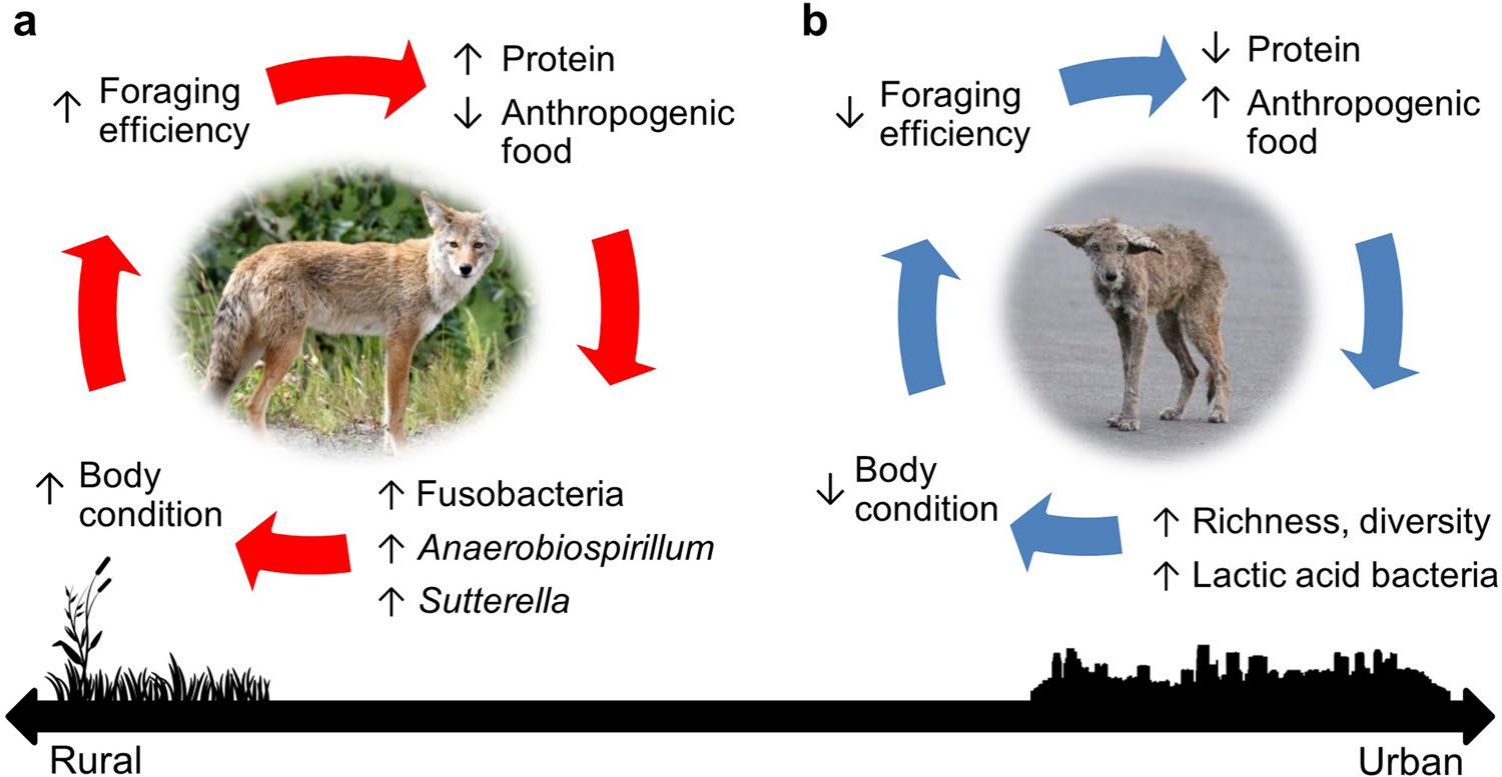
Conceptual model of positive feedback loops among diet, microbiome composition, and health. (**a**) Coyotes consuming more protein, and less anthropogenic food, have more Fusobacteria, *Anaerobiospirillum*, and *Sutterella* in their microbiome and improved body condition. This, in theory, promotes the ability to continue capturing prey, and is the more common cycle in rural environments. (**b**) Increased diet subsidization with anthropogenic food leads to increased microbiome richness and diversity, higher abundances of select lactic acid bacteria such as *Streptococcus* and *Enterococcus*, and lower body condition. Poor health likely decreases foraging efficiency, thus increasing the reliance on anthropogenic food. This cycle predominates in urban-exploiting coyotes (images from Jitze Couperus, *left*, and Michael Renzi, *right*).

We suspect that these directional associations linking an anthropogenic diet to poor health via microbiome diversity and LAB abundance introduce a microbial element into what has previously been described as a “vicious cycle” of diet, body condition, and disease susceptibility^53^ (Fig. 6a). Animals in poor condition are more likely to become diseased and are less successful at capturing prey, thus increasing their reliance on anthropogenic food. This cycle explains why diseased coyotes are reported more frequently at compost sites^37,38^. However, not all urban coyotes depend on anthropogenic food subsidies: while some urban-dwelling coyotes range freely through developed land and consume anthropogenic food, others remain in urban natural areas and have diets nearly indistinguishable from rural coyotes^37,54^. In our study, urban coyotes that were classified as rural in random forest models likely represent this latter group, as misclassified coyotes had lower alpha diversity measures, lower LAB abundances, and appeared to be slightly healthier. Although microbiome diversity and LAB are frequently cited as a benefit to the host^44^, their negative effects in coyotes speak to the importance of understanding the functional value of commonly used microbiome measures in different host species living in different environments.

A complementary positive feedback loop characterizing rural coyotes revolved around Fusobacteria, *Sutterella*, and *Anaerobiospirillum*, in which coyotes consuming more prey harbored more of these taxa in their microbiome and were generally healthier. Healthy coyotes could, in turn, be expected to have better success obtaining protein-rich prey (Fig. 6b). Fusobacteria are considered a core member of the canine microbiome, despite their low abundance in other mammals^55^ and association with disease and cancer in humans^56^. Our observation that Fusobacteria are positively correlated with protein consumption agrees with previous observations that dogs fed protein-rich diets harbored more Fusobacteria than dogs fed carbohydrate-rich kibble^57–60^. *Anaerobiospirillum* and *Sutterella* are among the most abundant Proteobacterial genera in canines^61^, including wolves^62^, and may also play key roles in protein digestion^61^. Other general indicators of health in our study, including *Alloprevotella, Allobaculum* and *Dorea*, have also been connected to health in canines^18,57,62^. In one study, *Alloprevotella* and *Sutterella* were detected in healthy dogs but were absent in dogs infected with canine parvovirus^63^.

The families *Erysipelotrichaceae, Coriobacteriaceae*, and *Lachnospiraceae* were the most consistent indicators of spleen mass, which we used as a proxy for immune system function. *Erysipelotrichiaceae* are associated with carbohydrate consumption in dogs^57,59^ and correlates with colonic inflammatory responses in both humans and mice^64^. In humans, *Coriobacteriaceae* has also been connected to the abundance of pro-inflammatory cytokines^65^, and *Lachnospiraceae*, specifically *Blautia*, have been shown to decrease after splenectomy^66^. Interactions between the microbiome and the immune system are complex, and the interpretive power provided by the coarse measure of spleen mass alone is low; large spleens have alternately been considered indicators of immune stress^67^ or immune strength^40^. While we hypothesized that larger spleens reflect immune stress due to their association with urban areas and carbohydrate-degrading taxa, our results warrant more detailed studies of how the microbiome may regulate an immune response in urban coyotes. However, our data provide further evidence of the “vicious cycle” feedback loop in two ways: (1) spleen mass grouped with anthropogenic food consumption in rCCA models; and (2) larger spleens predicted low short-term prey consumption in structural equation models. This cycle predicts that unhealthy coyotes are less able to obtain prey and become more dependent on anthropogenic food subsidization, which may apply particularly to coyotes drawn to compost^38^.

An important additional observation in our study is that many of the relationships between microbiome features and the recent consumption of anthropogenic food became weaker when evaluated using long-term δ^13^C signatures. Conversely, relationships with prey consumption were stronger when evaluated with the long-term measure δ^15^N. We hypothesize that this observation reflects a combination of natural constraints on the microbiome and the rapidity with which the microbiome responds to perturbation. Gut microbiome composition is limited by host gut physiology^31^, and coyotes naturally harbor a gut microbiome shared by other carnivores with short, simple guts^68^. Diet-induced changes in the microbiome can occur within hours of a meal and revert equally quickly to a stable state^69^. Anthropogenic meals represent a novel and transient perturbation to a stable carnivorous microbiome, whereas protein consumption reflects the natural state of a carnivore over extended time. Indeed, the lower NTI values in urban coyotes provide evidence for more competitive microbiome structuring as the microbiome frequently adjusts to changes in diet. This continuous competition or adjustment could exacerbate the vicious cycle described above, insofar as instability in the microbiome induced by frequent alterations among food sources may reduce natural barriers to infection and prevent the microbiome from adapting to or metabolizing the immediate diet^15^.

Other aspects of health and behavior may additionally be affected by the microbiome in unpredictable ways. Although we found no strong connection between microbiome composition and the presence of *E. multilocularis*, it remains possible that disrupted co-evolutionary dynamics between urban coyotes and their gut microbiota provide a fertile environment for parasite establishment^70^. Further investigations using more sensitive measures, such as absolute worm counts, are needed to explore parasite/microbiome relationships in more detail. Behavior may likewise be affected by the microbiome: one urban coyote in our study was lethally managed because it attacked and killed a large domestic dog, an uncharacteristically aggressive behavior for an urban coyote. Low-protein diets and poor health have been cited as predictors of conflict^35^, but this coyote was above average in all measures of condition and prey consumption. Instead, this was the only coyote that did not contain any detectable Fusobacteria in its fecal microbiome. Documented relationships between the microbiome and behavior are becoming increasingly common in humans and other animals^71^, and microbiome composition, including fewer Fusobacteria, has been directly associated with aggression in dogs^18^. Aggressive behavior and the spread of *E. multilocularis* both have great implications for human-coyote interactions in urban areas, and we suspect urban-induced disruptions to the microbiome may play important but poorly understood roles in mediating those outcomes.

## Conclusion

The urban landscape is highly heterogenous, and behavioral adaptations in urban animals generate a complex mosaic of spatially and temporally variable diets, lifestyle contexts, and environmental exposures among both individuals and species. Our results provide a foundational understanding of the gastrointestinal microbiome in urban-adapted coyotes and demonstrate how the consumption of carbohydrate-rich anthropogenic food alters a naturally carnivorous microbiome to negatively affect overall health. Moreover, we suggest mechanisms for how the microbiome participates in previously described positive feedback loops connecting diet, body condition, and disease susceptibility in urban animals. Further work may make it possible to use the bacterial taxa implicated in these feedback loops as indicators in non-invasive evaluations of coyote health via scat samples, providing similar information to urban wildlife managers as a dysbiosis index currently used for dogs in a veterinary setting^52^. We lastly speculate that urban-induced alterations in the microbiome may influence behavior and parasite susceptibility, though more work will be needed to untangle the exact mechanisms and magnitude of these relationships.

## Methods

### Sample collection and necropsy

We collected coyote carcasses from Edmonton (Alberta, Canada) and the surrounding area in the winters of 2017–2018 and 2018–2019. Samples were collected as roadkill, obtained from local fur trappers, or lethally managed after negative interactions with humans or domestic animals (Table S1). Coyotes were classified based on their location of death as either “urban” or “rural.” All samples, including roadkill, were collected and frozen within 24 h of death, and both collection seasons had similar temperature and precipitation patterns (see Supplementary Methods S1). Carcasses were stored at − 80 °C for 5 days to neutralize any zoonotic pathogens and then transferred to − 20 °C until necropsy.

At necropsy, we measured the mass, body size (snout to base of tail), and girth around the ribcage for each coyote, and we measured the kidney fat index (KFI) following published protocols^42^ and recorded the mass of the spleen. For analysis, spleen mass (in grams) was divided by body mass (in kilograms) to account for size differences among animals. We removed the lower mandible for age determination, clipped the left hind outer toenail for stable isotope analysis, and removed the stomach for dietary analysis. For microbiome analysis, we extruded fecal samples from the large intestine and stored them at − 80 °C.

### Age determination

We determined the age of each coyote by counting cementum annuli. Lower canine teeth were removed from the mandible by soaking the mandible at 80 °C for 6–8 h. Teeth were fixed in a neutral solution of 10% formalin before being decalcified, sectioned, and stained following published methods^72^. We followed the aging criteria reported by Linhart et al.^73^. To increase precision, we assigned all coyotes a birth date of 1 May and used the difference between birth date and death date to determine age to the nearest month.

### Stomach content analysis

To determine each coyote’s recent diet, we quantified stomach contents by volume. We rinsed stomach contents in an 850 μm sieve to remove mucus and then quantified the total food volume by measuring the amount of water displaced (in ml) when the contents were placed in a graduated cylinder. Discernable food items were then classified as prey (including rodents, small herbivores, ungulates, and insects), anthropogenic food (including fast food waste, compost, or other garbage), or vegetation (including grass, leaves, and sticks). The volume in each of these categories was measured using the same method as before.

### Stable isotope analysis

We used stable isotope values (δ^13^C and δ^15^N) measured from claw samples to infer each coyote’s habitual diet. Whole claw samples were rinsed three times with a 2:1 chloroform:methanol solution to remove residual lipids and surface oils and then dried at 37 °C for 5 days. After drying, we manually homogenized the distal 5 mm of each claw and weighed 1.5 mg subsamples into tin capsules. Samples were combusted using a Vario Pyrocube and analyzed using an Isoprime Vision Mass Spectrometer at the Biogeochemical Analytical Service Laboratory (Dept. of Biological Sciences, Univ. of Alberta).

Isotopic values were used in stable isotope mixing models to estimate the proportion of different food items in coyote diets. Stable isotope values for various coyote diet items were obtained from a previous study in our lab^37^, and diet items were categorized as prey, fruit, or anthropogenic food. Isotopic data for anthropogenic food was supplemented with published values for beef and chicken^74^. We accounted for tissue-specific discrimination factors following previously described methods^54^ and ran mixing models using the default settings of the R package *simmr*^75^.

### Microbiome analysis

We extracted total DNA from 100 mg of each fecal sample using the MP Bio FastDNA Spin Kit for Soil following the manufacturer’s instructions (MP Biomedicals, Santa Ana, CA)^68^. Fecal samples were thawed and manually homogenized using a pestle and mortar prior to extraction, and before the final elution we included a five-minute incubation at 50 °C to maximize DNA yield. Extracted DNA was submitted to Microbiome Insights (Vancouver, BC) for sequencing, where PCR amplification of the V4 region of the 16S rRNA gene was performed in 50 μl reactions with 2 μl of template DNA using previously described cycling conditions for the barcoded universal bacterial primers 515F and 806R^76^. Successful amplification was confirmed using agarose gel electrophoresis. Paired-end sequencing of equimolar concentrations of the PCR products was conducted on an Illumina MiSeq platform using V3 chemistry and 300 bp reads. All DNA extraction, PCR, and sequencing steps were performed alongside three negative control samples and a mock community (see Fig. S14).

Sequence data was processed to generate amplicon sequence variants, with taxonomic assignment and phylogenetic tree construction performed following a previously described protocol^68^ (see also Supplemental Methods). Five samples with fewer than 4,500 reads were excluded from downstream analysis, based on a visual examination of read count distributions across all samples and a sample completeness threshold of > 98% calculated using *iNext*^77^ (Fig. S13). In addition, we removed ASVs that were identified as chloroplasts, mitochondria, or protists in a BLAST search, as well as 21 putative contaminant ASVs detected using the prevalence-based detection method implemented in the package *decontam*^78^. No putative contaminant was either abundant (> 1% relative abundance) or prevalent (> 20% prevalence) across experimental samples (Fig. S13). These filtering procedures resulted in an average of 21,319 ± 9,477 reads per sample (range 4556–46,542).

### Parasite survey

We used PCR to test each coyote for possible infection with *E. multilocularis*. DNA extracted from fecal samples was amplified in triplicate using the *E. multilocularis*-specific primers Cest1 and Cest2^79^. PCR was performed in 25 μl reactions with 0.2 μM of each primer and 1 μl of template DNA using cycling conditions described previously^79^. We resolved PCR products using gel electrophoresis; a coyote was considered positive for *E. multilocularis* if the sample exhibited a 395 bp band in at least two replicates. Samples that tested negative were diluted and tested again to control for possible PCR inhibition.

### Statistical analyses

All statistical analyses were performed in R 3.6.2^80^ and a full description of our statistical workflow is available in the electronic supplementary material. In brief, a small number of missing physiological measurements were imputed using linear regressions. We then generated a single composite metric of physical condition by performing principal components analysis (PCA) on mass, body size, girth, and KFI and extracting the axis scores on the first principal component (Table S6). Spleen mass was retained as a separate measure of immune function because it did not load well on either of the first two components.

For each microbiome sample, we calculated total ASV richness, Shannon diversity, and Faith’s PD using *iNext* and *iNextPD*, which produce asymptotic estimates for these values based on rarefaction curves^77,81^. NTI was calculated using *picante*^82^. We used linear and logistic regressions to determine if any of our diet, microbiome, or condition measures varied with either coyote location or *E. multilocularis* infection status, while controlling for the effects of sex and age. Because there was a significant association between location and *E. multilocularis* infection, the effects of *E. multilocularis* infection were tested while also controlling for location.

To determine which measures were the best indicators of microbiome diversity, we used GLMs predicting each microbiome metric from the remaining diet and health measures. Continuous predictors were centered and standardized and all models subsets were examined. The relative importance of each predictor was determined by (1) summing the AIC model weights for each model in which the predictor appeared^83^ and (2) comparing model-averaged coefficients across models with a ΔAICc less than two. Model coefficients were standardized by their partial standard deviation prior to coefficient-averaging^84^.

We tested for differences in overall microbiome composition using three complementary approaches. First, differential abundance analyses were performed at all taxonomic levels using the ‘aldex.glm’ function in *ALDEx2*^85^ while controlling for confounding variables. We used the Benjamini–Hochberg correction to control for multiple comparisons and the Hedge’s *g* statistic as a measure of effect size. Second, random forest models were trained to predict coyote location from centered log ratio (CLR)-transformed taxon abundances, and we tested for any differences in diet and health between correctly and incorrectly classified urban coyotes using the regression methods described above. Third, we used an Aitchison distance-based PERMANOVA to test for differences in overall microbiome composition, and we mapped continuous diet and health measures as vectors onto a PCA of microbiome composition.

We then tested for relationships linking diet or health to the abundance of individual taxa, limiting our analyses to the 25 most prevalent bacterial genera with a mean relative abundance greater than 0.1%. Univariate associations were evaluated using Spearman’s correlation. For each taxon, we additionally constructed rank regression models, with either the condition index or spleen mass as a response variable and taxon abundance as the predictor. Sex was included as a covariate. We did not control for age in these models because, in wild systems, only animals in good condition live to be old, and age and health were therefore considered redundant measures (Fig. S15). Multivariate associations were further investigated using rCCA. We used correlation distance-based hierarchical clustering to identify taxa that responded similarly to the various explanatory variables. Taxon abundances were CLR-transformed for all analyses.

Because of the inter-correlated nature of our variables, we lastly used structural equation modeling (SEM) to test for causal linkages among variables. Where necessary, variables included in each model were log-transformed or scaled to meet the model assumptions. For each taxon cluster identified in the rCCA, we constructed initial models that represented general hypotheses of causal linkages among variables and we specified residual correlations among all microbiome features. Additional paths were added as recommended by modification indices and non-significant paths (p > 0.1) were removed. The final models were selected when adding or removing an additional path either caused model AIC to increase or caused other fit parameters to exceed conventional thresholds, even if the path was non-significant.

## Supplementary Information

Supplementary Information

## Data Availability

The raw sequencing data supporting the conclusions of this article have been deposited in the NCBI Short Read Archive under project number PRJNA528764. The R scripts and workspace required to reproduce all analyses and figures are available in the GitHub repository https://github.com/sasugden/Coyote_microbiome.
