# Supplementary material for: An altered microbiome in urban coyotes mediates relationships between anthropogenic diet and poor health
Source: Sci Rep. 2020 Dec 17;10:22207. doi: 10.1038/s41598-020-78891-1 (PMC7746695; doi:10.1038/s41598-020-78891-1)
Supplement: Supplementary file 1 — Supplementary Information [file 41598_2020_78891_MOESM1_ESM.pdf]

SUPPLEMENTARY MATERIAL for

An altered microbiome in urban coyotes mediates relationships between anthropogenic diet and poor health

Scott Sugden[a]*, ssugden@ualberta.ca ORCID: 0000-0002-6482-4733

Dana Sanderson[b], sandersond2@macewan.ca

Kyra Ford[a], kmford@ualberta.ca

Lisa Y. Stein[a], stein1@ualberta.ca

Colleen Cassady St. Clair[a], cstclair@ualberta.ca

[a] Department of Biological Sciences, University of Alberta, Edmonton, Alberta, Canada

[b] Department of Biological Sciences, MacEwan University, Edmonton, Alberta, Canada

* Author to whom correspondence should be addressed:

Scott Sugden

CW405 Biological Sciences Building

University of Alberta

Edmonton, AB T6G 2E9

(612) 584-9813

ssugden@ualberta.ca or sasugden@gmail.com

SUPPLEMENTARY METHODS

This section provides detailed explanations of select laboratory methods as well as the statistical procedures and parameters that were used for data analysis. The R workspace and script required to reproduce all analyses can be found at the link below:

https://github.com/sasugden/Coyote_microbiome

Sample collection

Samples were collected in the winters of 2017-2018 and 2018-2019, with all but three samples collected between October and April (inclusive) of each year. Road-killed carcasses were obtained from the City of Edmonton Animal Care and Control Centre, which retrieves roadkill within 24 hours of death. Trapped carcasses were provided by Animal Damage Control, which ran fur trapping lines that were checked daily throughout the study period. Lethally managed coyotes were collected at the moment of death by the City of Edmonton Police Service. All samples were therefore obtained and frozen within 24 hours of death. In addition, average monthly temperatures ranged from -19.4°C to 4.6°C during our sampling period, with average temperatures above freezing only in October and April. Samples would have begun freezing naturally soon after death. Both winters were also marked by similar maximum snow depths (34 cm in 2017-2018 and 37 cm in 2018-2019). Later analysis confirmed that there were no detectable differences in microbiome composition associated with collection season or sample preservation (**Fig. S16**).

DNA extraction and sequencing

All DNA extractions were performed manually in single tubes. This approach has been shown to reduce the risk of well-to-well contamination in sequencing experiments[1]. We tested extracted DNA samples for purity using a Nanodrop spectrophotometer and quantified yield using Qubit 1.0 (Thermo-Fisher) before submitting samples for sequencing. All coyote samples were submitted on a single 96-well plate with one negative control; the remaining control samples were submitted on separate plates.

Data processing

All statistical analyses were performed in R 3.6.2[2]. There were a small number of cases where a coyote was missing a physiological measurement; for example, two coyotes were decapitated for rabies testing prior to sample acquisition, so body mass and length data could not be measured. In these cases, missing data were imputed using linear regressions constructed with data from the remaining samples, with available physiological measures as predictors. The composite health index was generating using principal components analysis implementing using the 'rda' function in the R package *vegan*[3]. Axis scores on the first principal component explained 83.7% of variation among individuals and were used as a single index of physical condition (**Table S6**).

16S rRNA sequence data was processed to generate amplicon sequence variants (ASVs) using the R package *dada2*[4] following a previously described method[5]. In brief, forward and reverse reads were truncated at 240 bp and 160 bp, respectively, and low-quality reads were removed using the *dada2* default filtering parameters. Because *dada2* evaluates error rates for an entire sequencing run, and is therefore more accurate when more samples from the same run are evaluated together, the 16S rRNA sequence data described in this experiment was processed in *dada2* alongside data from concurrently sequenced experiments in our lab. The sequence data from separate experiments was removed immediately after the *dada2* processing pipeline.

Five coyote fecal samples with fewer than 4,500 reads were excluded from downstream analysis; this cutoff threshold was determined based on a visual examination of our read count distributions across all samples and supplemented by an evaluation of sample completeness measures calculated using *iNext*[6]. ASVs were then aligned against taxa in the RDP reference database (release 11.5)[7] using the naïve Bayesian classifier method implemented in *dada2*[8]. We removed 11 ASVs that were identified as chloroplasts or mitochondria, as well as 21 ASVs identified as contaminants using a prevalence-based detection threshold of 0.25 in the R package *decontam*[9], and confirmed that contaminants were neither abundant nor prevalent in our experimental data (**Fig. S13**). We then used the package *phangorn*[10] to generate a generalized time-reversible maximum likelihood phylogenetic tree for our data following previously described procedures[11]. The resulting feature table, taxonomic information, and phylogenetic tree were imported into the package *phyloseq*[12] for analysis.

For all downstream analyses, we present results obtained using an unrarefied, centered log ratio (CLR)-transformed feature table, as the CLR transformation best accounts for the compositional nature of microbiome data[13]. Total ASV richness, Shannon diversity, and Faith's phylogenetic diversity were determined as asymptotic estimates calculated from rarefaction curves implemented in the packages *iNext*[6] and *iNextPD*[14], and the nearest taxon index (NTI) was calculated using the package *picante*[15]. To ensure that our results were robust to the analysis method we chose, we additionally averaged ASV abundances across 1,000 rarefactions to the minimum remaining library size of 4,559 reads. Alpha- and beta-diversity analyses were repeated using this rarefied feature table, with alpha diversity measures calculated directly from rarefied data rather than estimated from rarefaction curves.

Regression models

We used linear regressions to determine if any of our measures of body condition, diet, or microbiome richness or diversity varied significantly between urban and rural coyotes, while controlling for any effects of sex and age. In these analyses, stomach content volumes were natural log-transformed with a pseudocount of 0.01 ml to meet the assumptions of a normal distribution. Significance (Type II ANOVA) was measured as $p<0.05$. The effects of sex, age, and location on *E. multilocularis* infection status were similarly evaluated using a logistic regression. Because there was a significant association between *E. multilocularis* infection and coyote location, the effects of *E. multilocularis* infection on body condition, diet, and microbiome alpha diversity were tested while controlling for location in addition to age. All regression models were implemented using the 'lm' and 'glm' functions in R, with the exception of models predicting ASV richness, which was fit with a negative binomial distribution with the 'glm.nb' function in the R package *MASS*[16].

To determine which measures were the best indicators of the four microbiome metrics (richness, Shannon diversity, Faith's PD, and NTI), we used GLMs with the microbiome metric as the response variable and stable isotope measures, stomach contents, *E. multilocularis* infection status, the physical condition index, and spleen mass as predictors. Continuous predictors were centered and standardized prior to model construction, and we confirmed that no predictor had a variance inflation factor greater than two. We examined all models subsets using the package *MuMIn*[17] and evaluated the relative importance of each predictor by 1) summing the

AIC model weights for each model in which the predictor appeared[18] and 2) comparing model-averaged coefficients across models with a ΔAICc less than two. To control for even slight collinearity among predictors, we standardized predictor coefficients by the partial standard deviation of their variables before averaging coefficients across top-ranked models, following the recommendations of Cade (2015)[19].

Microbiome community analyses

Differential abundance analyses were performed at the phylum, class, order, family, and genus levels using the R package *ALDEx2*[20]. Tests were performed using the 'aldex.glm' function and a pre-specified model matrix to control for confounding variables. Specifically, differential abundance was tested based on 1) sex, controlling for the effect of age; 2) location, controlling for the effects of sex and age; and 3) *E. multilocularis* infection status, controlling for the effects of location.

Random forest models trained to predict coyote location from CLR-transformed taxon abundances were implemented using the R package *randomForest*[21]. Models were run with 1,000 trees and included only ASVs with an average relative abundance greater than 0.01%.

We used an Aitchison distance-based permutational multivariate analysis of variance (PERMANOVA) with 1,000 permutations to test for differences in overall microbiome composition due to sex, age, location, or *E. multilocularis* infection. This analysis was implementing using the 'adonis' function in the R package *vegan*. We also mapped continuous diet and health measures onto a principal component analysis using the 'envfit' function in *vegan*. These analyses were repeated using the Bray-Curtis and Jaccard distance calculated from rarefied data and using the weighted and unweighted UniFrac distances[22] to test for potential phylogenetic clustering effects.

Relationships with taxon abundances

Multivariate associations among CLR-transformed abundances, short- and long-term diet measures, the physical condition index, spleen mass, and age were investigated using regularized canonical correlation analysis (rCCA) with three components, implemented in the package *mixOmics*[23]. We used correlation distance-based hierarchical clustering on the resulting similarity matrix to identify taxa that responded similarly to the various explanatory variables

and determined the importance of individual taxa based on the sum of the absolute values of their relevance scores.

Structural equation models were implemented in the R package *lavaan*[24]. Models were designed to test for causal linkages among location, diet, taxon abundances, physical condition score, and spleen mass. Where necessary, variables included in each model were log-transformed or scaled to meet the assumptions of homogenous variance and a normal distribution: the volumes of prey and anthropogenic food in the stomach were log-transformed after the addition of a pseudocount of 0.01 ml and then rescaled, and ASV richness was rescaled without transformation. To maintain a minimum 10:1 ratio of variables to observations, we limited models to nine variables. For each taxon cluster identified in the rCCA, we constructed initial models for evaluation that represented general hypotheses of causal linkages among variables, and we specified residual correlations among all microbiome features. Additional paths were added to the models as recommended by modification indices and non-significant paths ($p>0.1$) were removed. The final models were selected when adding or removing an additional path either caused model AIC to increase or caused other fit parameters to exceed conventional thresholds, even if the path was non-significant. Model coefficients were standardized in the top models to facilitate comparison among paths.

REFERENCES

1. Minich, J. J. *et al.* Quantifying and understanding well-to-well contamination in microbiome research. *mSystems* **4**, 1–13 (2019).
2. R Core Team. R: A language and environment for statistical computing. (2019).
3. Oksanen, J. *et al.* vegan: Community Ecology Package. (2018).
4. Callahan, B. J. *et al.* DADA2: High-resolution sample inference from Illumina amplicon data. *Nat. Methods* **13**, 581–583 (2016).
5. Sugden, S. A., St. Clair, C. C. & Stein, L. Y. Individual and site-specific variation in a biogeographical profile of the coyote intestinal microbiota. *Microb. Ecol.* (2020).
6. Hsieh, T. C., Ma, K. H. & Chao, A. iNEXT: an R package for rarefaction and extrapolation of species diversity (Hill numbers). *Methods Ecol. Evol.* **7**, 1451–1456 (2016).
7. Cole, J. R. *et al.* Ribosomal Database Project: Data and tools for high throughput rRNA analysis. *Nucleic Acids Res.* **42**, 633–642 (2014).
8. Wang, Q., Garrity, G. M., Tiedje, J. M. & Cole, J. R. Naive Bayesian classifier for rapid assignment

of rRNA sequences into the new bacterial taxonomy. *Appl. Environ. Microbiol.* **73**, 5261–5267 (2007).

9. Davis, N. M., Proctor, D. M., Holmes, S. P., Relman, D. A. & Callahan, B. J. Simple statistical identification and removal of contaminant sequences in marker-gene and metagenomics data. *Microbiome* **6**, 226 (2018).
10. Schliep, K. P. phangorn: phylogenetic analysis in R. *Bioinformatics* **27**, 592–593 (2011).
11. Callahan, B. J., Sankaran, K., Fukuyama, J. A., McMurdie, P. J. & Holmes, S. P. Bioconductor workflow for microbiome data analysis: from raw reads to community analyses. *F1000Research* **5**, 1492 (2016).
12. McMurdie, P. J. & Holmes, S. phyloseq: An R package for reproducible interactive analysis and graphics of microbiome census data. *PLoS One* **8**, e61217 (2013).
13. Gloor, G. B., Macklaim, J. M., Pawlowsky-Glahn, V. & Egozcue, J. J. Microbiome datasets are compositional: And this is not optional. *Front. Microbiol.* **8**, 2224 (2017).
14. Hsieh, T. C. & Chao, A. iNextPD: interpolation and extrapolation for phylogenetic diversity. (2017).
15. Kembel, S. *et al.* Picante: R tools for integrating phylogenies and ecology. *Bioinformatics* **26**, 1463–1464 (2010).
16. Venables, W. N. & Ripley, B. D. *Modern Applied Statistics with S*. (Springer, 2002).
17. Barton, K. MuMIn: Multi-Model Inference. (2018).
18. Giam, X. & Olden, J. D. Quantifying variable importance in a multimodel inference framework. *Methods Ecol. Evol.* **7**, 388–397 (2016).
19. Cade, B. S. Model averaging and muddled multimodel inferences. *Ecology* **96**, 2370–2382 (2015).
20. Fernandes, A., Macklaim, J. M., Linn, T., Reid, G. & Gloor, G. B. ANOVA-like differential expression (ALDEx) analysis for mixed population RNA-Seq. *PLoS One* **8**, e67019 (2013).
21. Liaw, A. & Wiener, M. Classification and regression by randomForest. *R News* **2**, 18–22 (2002).
22. Lozupone, C. & Knight, R. UniFrac: A new phylogenetic method for comparing microbial communities. *Appl. Environ. Microbiol.* **71**, 8228–8235 (2005).
23. Rohart, F., Gautier, B., Singh, A. & Le Cao, K.-A. mixOmics: An R package for ‘omics feature selection and multiple data integration. *PLoS Comput. Biol.* **13**, e1005752 (2017).
24. Rosseel, Y. lavaan: An R package for structural equation modeling. *J. Stat. Softw.* **48**, 1–36 (2012).

SUPPLEMENTARY TABLES & FIGURES

Table S1: Summary of coyotes included in this study.

Numbers indicate the numbers of coyotes collected from each location, sex, and source. "Managed" refers to coyotes that were lethally managed due to conflict or conflict-prone behavior.

	Urban		Rural		*Totals*
	M	F	M	F	
Trapped	0	0	34	30	**64**
Roadkill	14	11	0	0	**25**
Managed	3	2	1	0	**6**
Totals	**17**	**13**	**35**	**30**	**95**

Table S2: Sex-based differences among samples.

The mean and standard deviation of all measures of body condition, diet, and microbiome diversity are shown for each sex. For statistical comparisons, see Table 1 in the main manuscript.

	Mean Value				
	Male	Female	t	df	p
Mass (kg)	11.71	9.42	-5.40	93	**< 0.001**
Body size (cm)	88.58	84.07	-3.64	93	**< 0.001**
Girth (cm)	48.34	44.30	-4.15	93	**< 0.001**
Age (yr)	2.85	1.81	-2.31	93	**0.023**
Spleen size (g/kg)	1.87	1.94	0.53	93	0.597
KFI	0.54	0.44	-1.55	93	0.124
Robustness index	0.04	-0.05	-4.36	93	**< 0.001**
$\delta^{13}C$ (‰)	-22.42	-22.49	-0.31	93	0.757
$\delta^{15}N$ (‰)	8.97	8.75	-1.55	93	0.125
Vol. anthro food (ml)	22.51	33.99	0.71	93	0.479
Vol. prey (ml)	174.31	150.87	-0.75	93	0.456
Diet diversity	0.27	0.27	0.02	93	0.948
ASV Richness	120.61	138.95	1.49	86	0.139
Shannon index	2.74	2.83	0.55	86	0.585

Table S3: ANOVA results evaluating the effects of sex, age, and habitat use on the prevalence of dietary items and *E. multilocularis*.

The prevalence of dietary items and *E. multilocularis* was modeled using a logistic regression with sex, age, and habitat use as predictors. Significance of each term was tested using an ANOVA.

	Prevalence (%)		Sex			Age			Location		
	Urban	Rural	F	df	p	F	df	p	F	df	p
Empty stomach	20	13.8	0.11	1	0.746	0.67	1	0.413	0.80	1	0.371
Anthropogenic food	50	53.8	0.00	1	0.992	3.63	1	0.057	0.32	1	0.572
Natural prey	84.6	70	1.44	1	0.230	0.00	1	0.948	2.88	1	0.090
Vegetation	80	76.9	0.20	1	0.657	2.26	1	0.133	0.22	1	0.638
E. multilocularis	53.3	35.4	0.60	1	0.438	6.57	1	**0.010**	2.27	1	0.132

Table S4: PERMANOVA results evaluating the effects of age, sex, and habitat use on microbiome composition.

Permutational multivariate analyses of variance were performed with 1,000 permutations.

	PERMANOVA			
Variable	df	F	R^2	p
**Aitchison**				
Sex	1	1.091	0.012	0.233
Age	1	1.830	0.020	**0.003**
Location	1	2.831	0.032	**0.001**
**Bray-Curtis**				
Sex	1	1.044	0.012	0.344
Age	1	1.450	0.016	0.103
Location	1	4.026	0.044	**0.001**
**Jaccard**				
Sex	1	1.059	0.012	0.311
Age	1	1.373	0.015	**0.001**
Location	1	2.752	0.031	**0.001**
**Weighted UniFrac**				
Sex	1	1.445	0.016	0.181
Age	1	1.324	0.014	0.204
Location	1	4.856	0.053	**0.002**
**Unweighted UniFrac**				
Sex	1	1.226	0.013	0.186
Age	1	2.273	0.025	**0.005**
Location	1	4.213	0.046	**0.001**

Table S5: Structural equation model path and fit statistics.

Three separate structural equation models were run testing for predicted relationships among habitat use, diet, microbiome composition, health, and *E. multilocularis* infection status. Taxa were selected for these models based on the results of a regularized canonical correlation analysis (rCCA). Models were run based on based on taxa implicated for their relationships with (1) anthropogenic food consumption; (2) protein consumption; and (3) spleen mass. From the proposed models, non-significant paths were removed until the removal of an additional path caused the model AIC to increase. For each model, the model chi-squared, degrees of freedom (df), and p-value are provided, along with the standardized root mean residual (SRMR), root mean square error of approximation (RMSEA), comparative fit index (CFI), and non-normed fit index (NNFI). In addition, R^2 values for each endogenous variable and standardized coefficients and p-values for each path are shown.

χ^2	11.067	SRMR	0.047
df	19	RMSEA	0
p	0.922	CFI	1
		NNFI	1.118

R^2	Response	Predictor	Coef.	p
7.2	**Health**			
		Streptococcus	-0.194	0.075
		ASV Richness	-0.261	0.017
27.5	**Spleen mass**			
		Location	0.494	< 0.001
		E. multilocularis	-0.166	0.068
		Enterococcus	-0.157	0.083
34.1	**δ^{13}C**			
		Location	0.584	< 0.001
33.9	**ASV Richness**			
		Vol. anthro	0.1	0.169
		δ^{13}C	-0.105	0.272
		Location	0.632	< 0.001
2.7	**Streptococcus**			
		Vol. anthro	0.165	0.018
9.3	**Enterococcus**			
		Vol. anthro	0.305	0.003
5.0	***E. multilocularis***			
		Location	0.223	0.032

χ^2	14.477	SRMR	0.067
df	19	RMSEA	0
p	0.755	CFI	1
		NNFI	1.060

R^2	Response	Predictor	Coef.	p
6.7	**Health**			
		Location	-0.185	0.076
		δ^{15}N	0.155	0.137
26.1	**Spleen mass**			
		Location	0.463	< 0.001
		E. multilocularis	-0.107	0.281
		Anaerobiospirillum	-0.231	0.054
		Sutterella	0.307	0.011
2.2	**δ^{15}N**			
		Location	-0.148	0.162
11.6	**Vol. prey**			
		Spleen mass	-0.331	0.001
11.5	**Fusobacterium**			
		Location	-0.190	0.014
		δ^{15}N	0.254	0.012
6.0	**Anaerobiospirillum**			
		δ^{15}N	0.246	0.017
4.6	**Sutterella**			
		δ^{15}N	0.213	0.041
8.4	***E. multilocularis***			
		Location	0.271	0.009
		Vol. prey	0.108	0.316

χ^2	6.87915	SRMR	0.036
df	16	RMSEA	0
p	0.97552	CFI	1
		NNFI	1.110

R^2	Response	Predictor	Coef.	p
8.0	**Health**			
		Location	-0.291	0.009
		Lachnospiraceae	0.210	0.058
23.6	**Spleen mass**			
		Location	0.439	< 0.001
		E. multilocularis	-0.151	0.113
		Erysipelotrichaceae	0.121	0.232
34.1	**δ^{13}C**			
		Location	0.584	< 0.001
17.7	**Erysipelotrichaceae**			
		Location	0.400	< 0.001
		Vol. anthro	0.138	0.073
12.2	**Coriobacteriaceae**			
		Location	0.335	0.001
		Vol. anthro	-0.093	0.219
15.6	**Lachnospiraceae**			
		Location	0.395	< 0.001
6.9	***E. multilocularis***			
		Location	0.316	0.013

Table S6: Pearson's correlation coefficients between principal component analysis axis scores and the variables used in the analysis.

Principal components analysis (PCA) was performed for mass, body size, girth, and the kidney fat index (KFI). The first principal component represented all four variables and explained 83.7% of among-sample variation, and was therefore used as a single composite index of health for downstream statistical analyses.

	PC1 (83.7%)		PC2 (14.4%)	
	R	p	R	p
Mass (kg)	0.839	**< 0.001**	-0.280	**0.006**
Body size (cm)	0.949	**< 0.001**	0.315	**0.002**
Girth (cm)	0.876	**< 0.001**	-0.477	**< 0.001**
KFI	0.307	**0.002**	-0.265	**0.010**

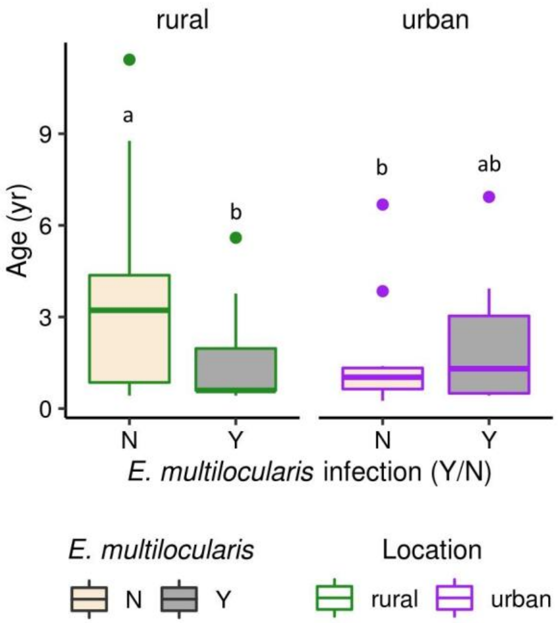

Fig. S1: Relationship between coyote age and *E. multilocularis* infection.

Coyotes infected with *E. multilocularis* are more likely to be younger. This trend is more evident in rural coyotes. Letters indicate significant pairwise differences among samples (Tukey's *post hoc* test, $p<0.05$).

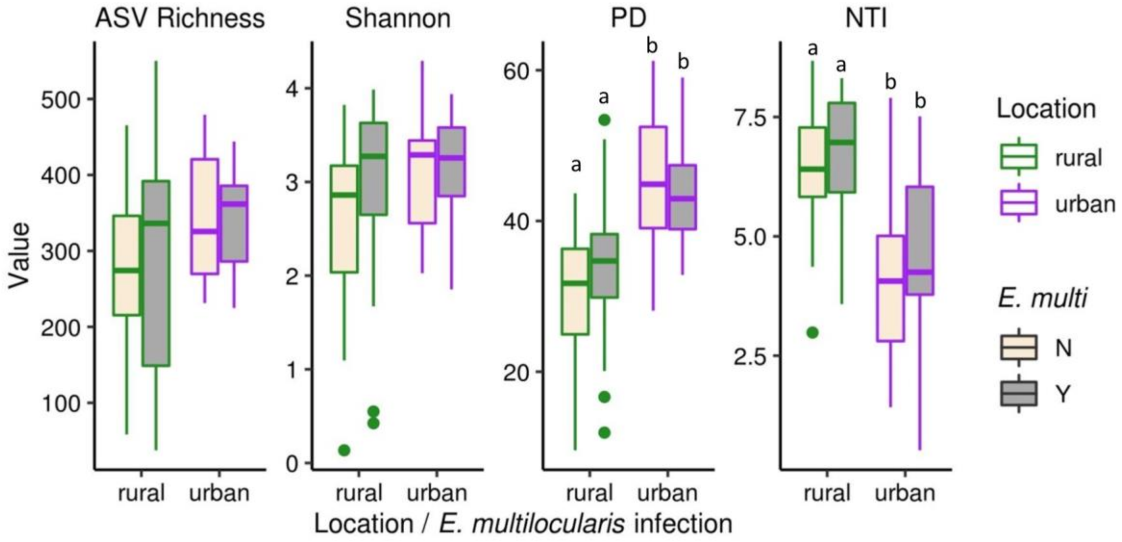

Fig. S2: Effects of E. *multilocularis* infection on microbiome alpha-diversity in urban and rural coyotes.

Coyotes infected with *E. multilocularis* had increased ASV richness, Shannon diversity, and phylogenetic diversity in their fecal microbiome. This effect was more pronounced in rural coyotes; urban coyotes already had higher values for these measures, and *E. multilocularis* infection did not further increase them. Letters indicate significant pairwise differences among samples (Tukey's *post hoc* test, p<0.05).

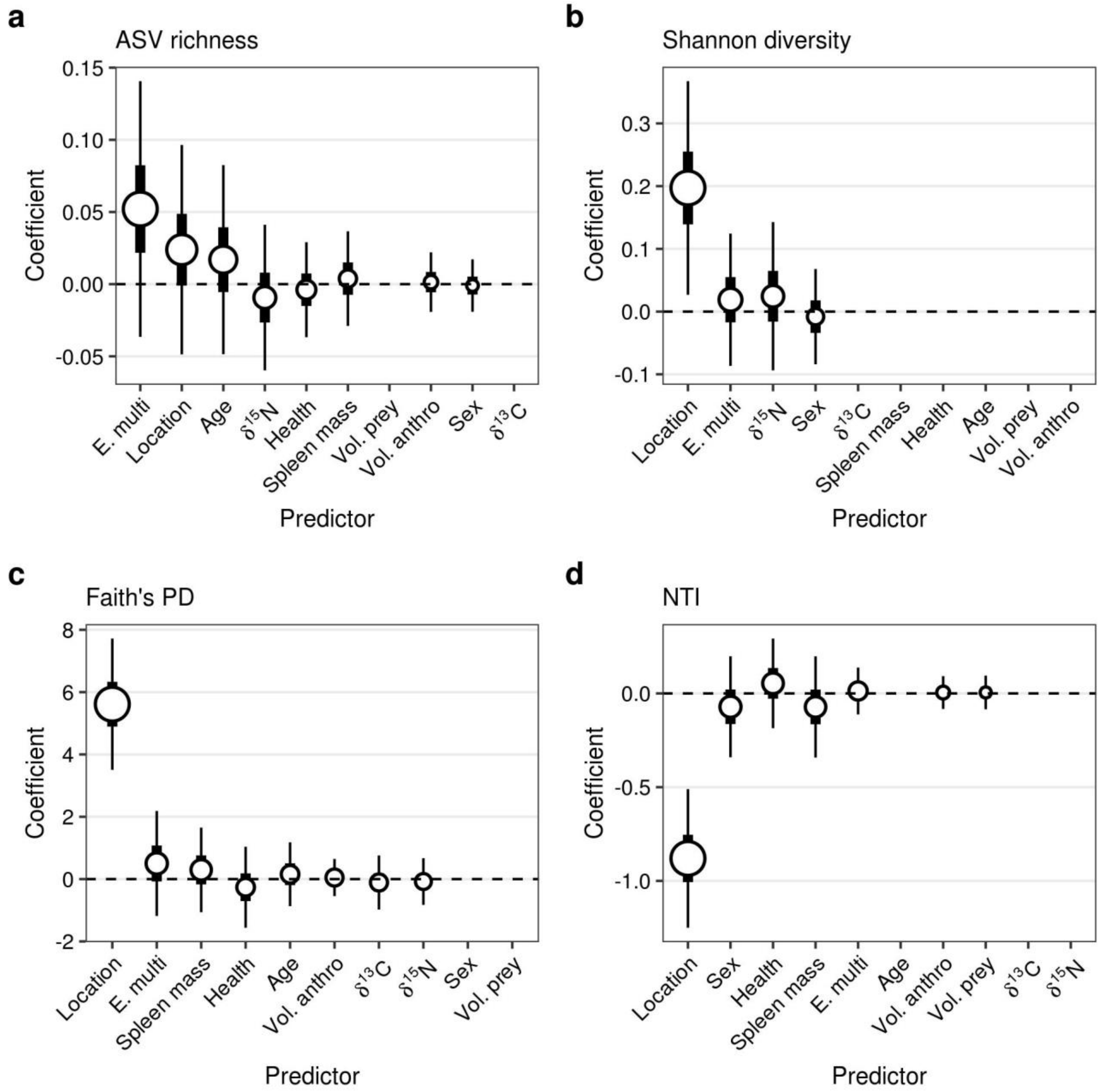

Fig. S3: Strongest predictors of microbiome alpha-diversity.

(**a**) ASV richness, (**b**) Shannon diversity, (**c**) Faith's phylogenetic diversity, and (**d**) the nearest taxon index (NTI) were each modeled as a function of measures of coyote habitat use, *E. multilocularis* infection, diet, and health. All possible model subsets were considered. Coefficients (y-axes) were averaged across all top-ranked models ($\Delta AIC_C < 2$) after being standardized by their partial standard deviation, and predictors were ordered along the x-axis based on the sum of the AIC model weights of the models in which they appeared (size of points). No coefficients are shown for predictors that did not appear in the top-ranked models.

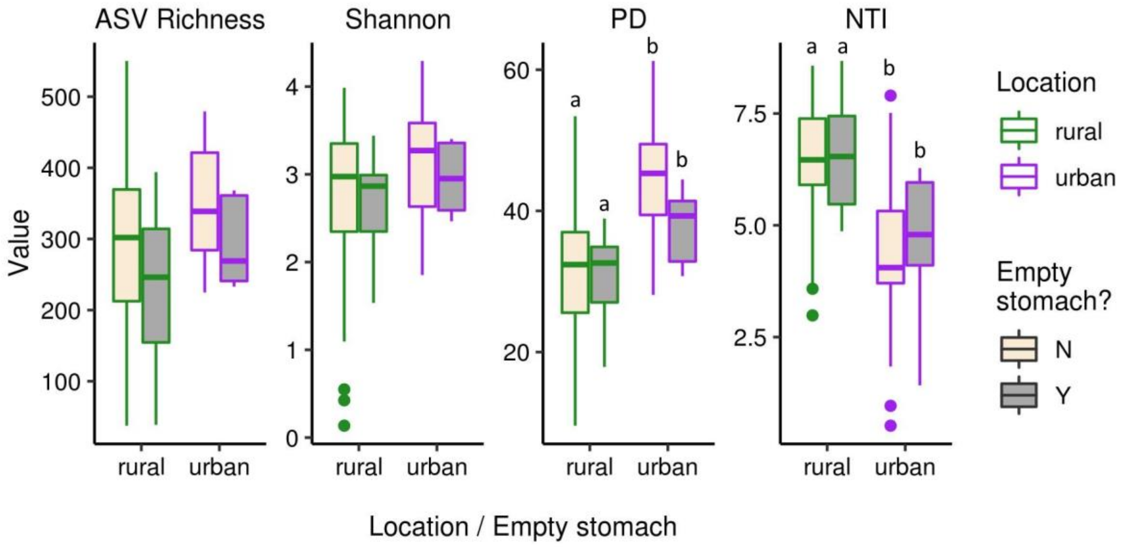

Fig. S4: Effects of an empty stomach on alpha diversity in urban and rural coyotes. Coyotes with no food in their stomachs exhibited lower ASV richness, Shannon diversity, and Faith's phylogenetic diversity (PD) than coyotes with evidence of a recent meal. There was limited effect on the nearest taxon index (NTI). This effect was evident in both urban and rural coyotes. Letters indicate significant pairwise differences among samples (Tukey's *post hoc* test, $p<0.05$).

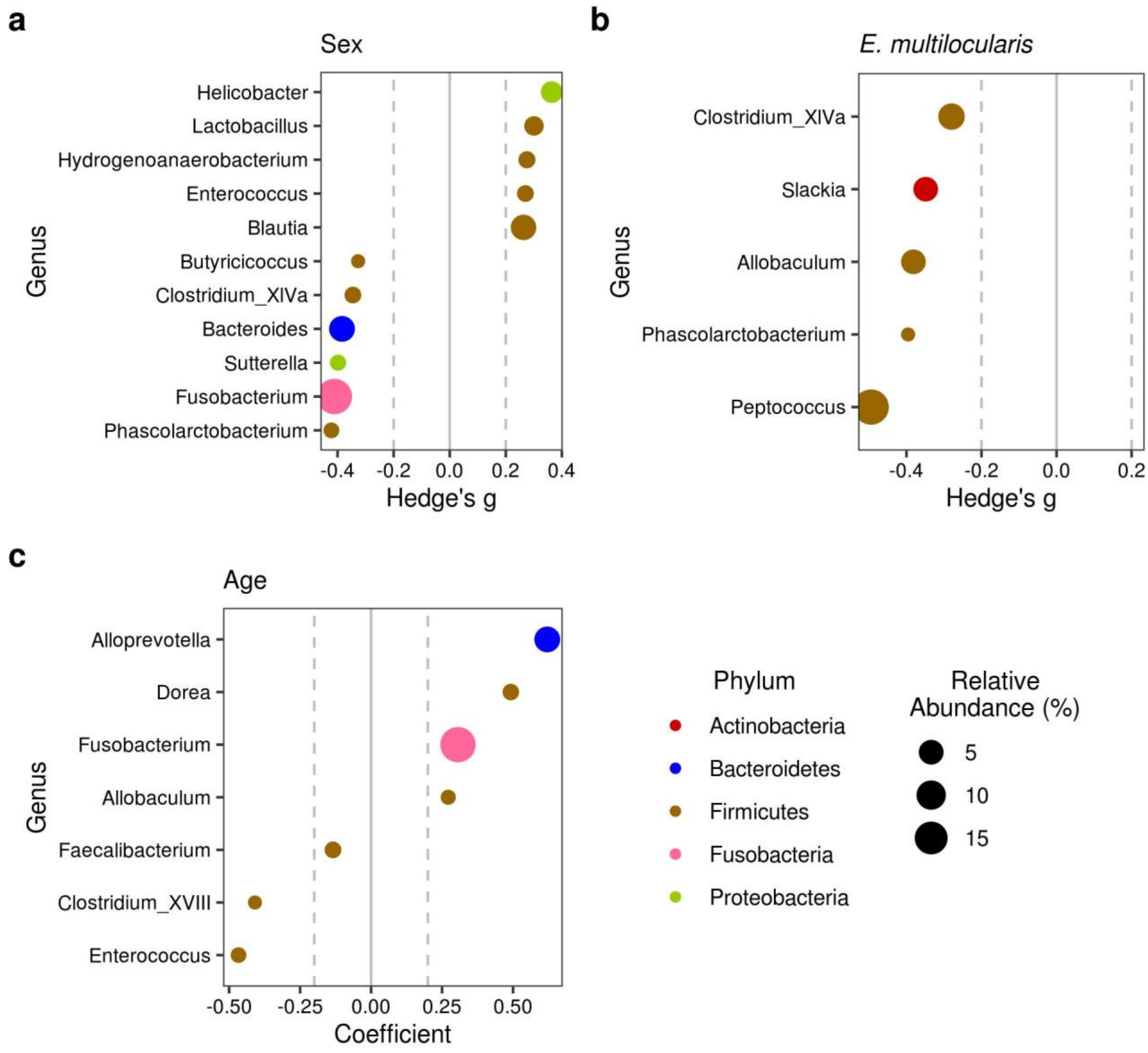

Fig. S5: Differential abundance based on sex, age, or *E. multilocularis* infection

Differential abundance was tested using *ALDEx2* for (**a**) sex, controlling for age; (**b**) *E. multilocularis* infection status, controlling for location; and (**c**) age, controlling for sex. In (**a**) and (**b**), genera are ranked by the Hedge's *g* measure of effect size between groups. In (**c**), genera are ranked by their coefficient in the generalized linear model produced by *ALDEx2*. No taxa were significantly differentially abundant ($p<0.05$) after the Benjamini-Hochberg adjustment for multiple corrections.

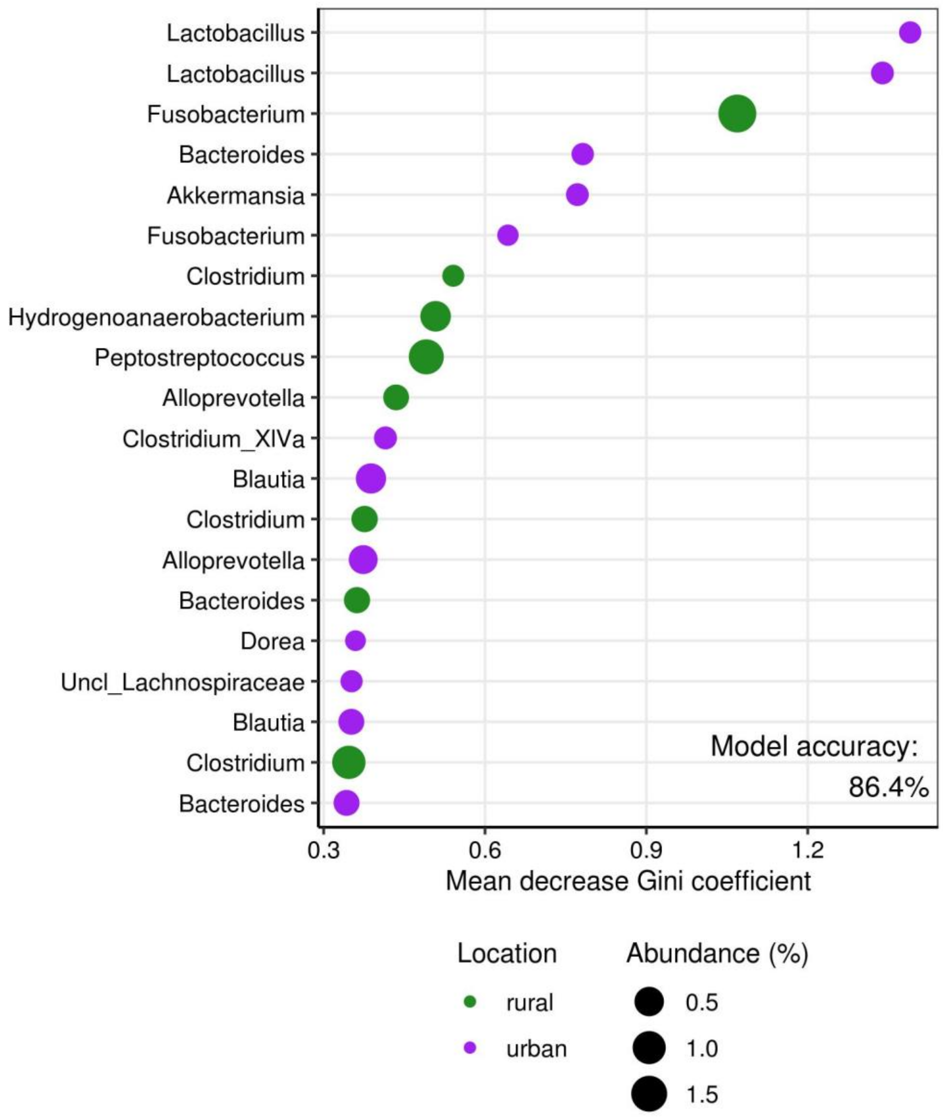

Fig. S6: Discriminatory ASVs in a random forest model predicting coyote habitat use. A random forest model was trained to predict coyote habitat use based on fecal microbiome composition. Discriminatory features (ASVs) were ranked by their mean decrease in the Gini coefficient. Point size indicates mean relative abundance (%) and colors indicate whether the ASV was more abundant in rural (green) or urban (purple) coyotes.

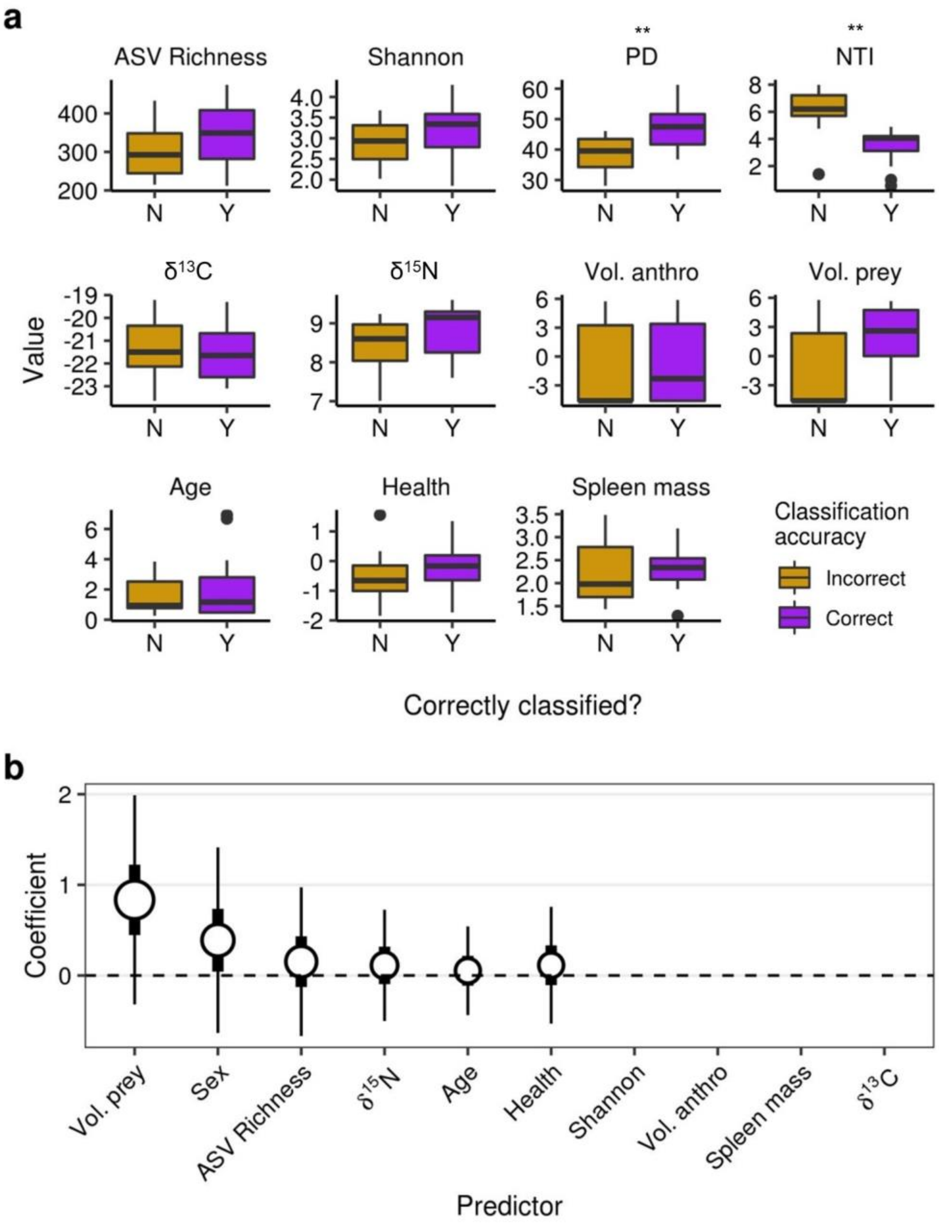

Fig. S7: Differences between correctly and incorrectly classified coyotes

(**a**) Diet, health, and microbiome measures were compared between urban coyotes that were correctly classified as urban in the random forest models and urban coyotes that were classified as rural. Comparisons where $p<0.05$ are indicated by asterisks (**). (**b**) In addition, classification accuracy was predicted in a logistic regression model using the same diet, health, and microbiome measures. Predictor coefficients were averaged across all model subsets with $\Delta AIC_C < 2$ and predictors were ranked based on the sum of the weights of the models in which they appeared.

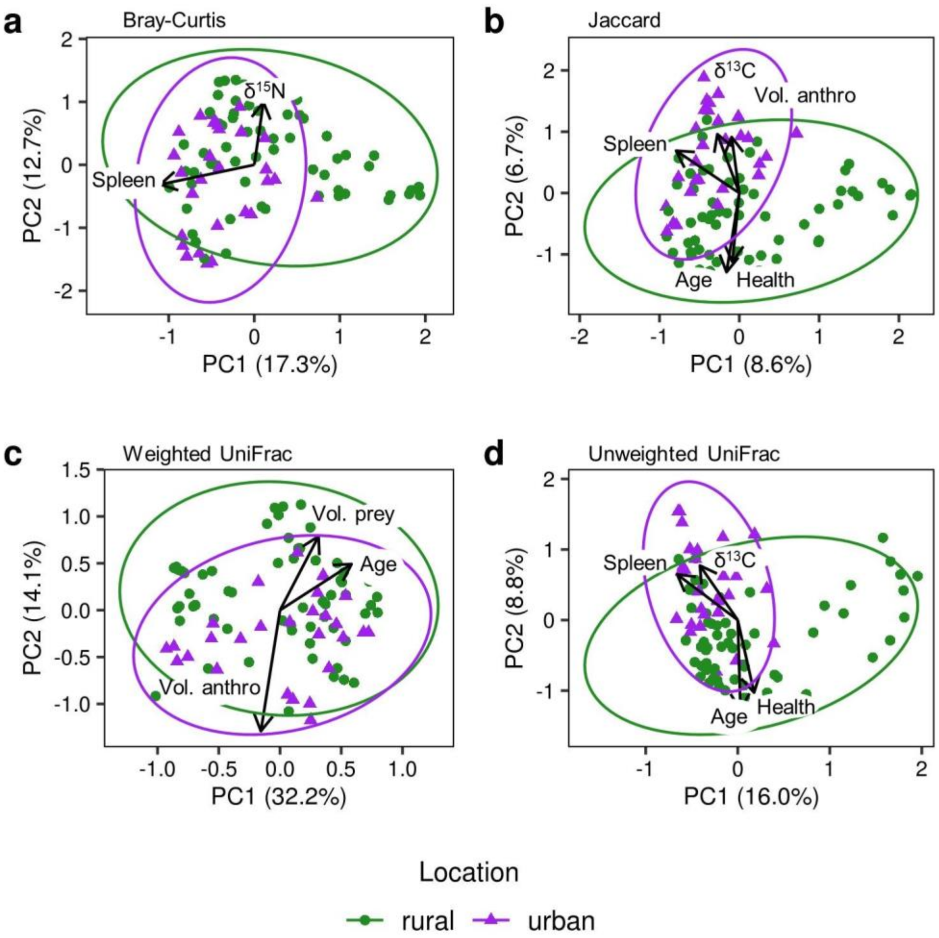

Fig. S8: Alternative distance metrics for evaluating coyote microbiome composition. Effects of location, diet, and health and microbiome composition were tested using the (**a**) Bray-Curtis, (**b**) Jaccard, (**c**) weighted UniFrac, and (**d**) unweighted UniFrac distance metrics calculated from rarefied data. Vectors were fit to principal coordinate analyses using the *envfit* function in the R package 'vegan,' and only significant vectors ($p<0.05$) are shown.

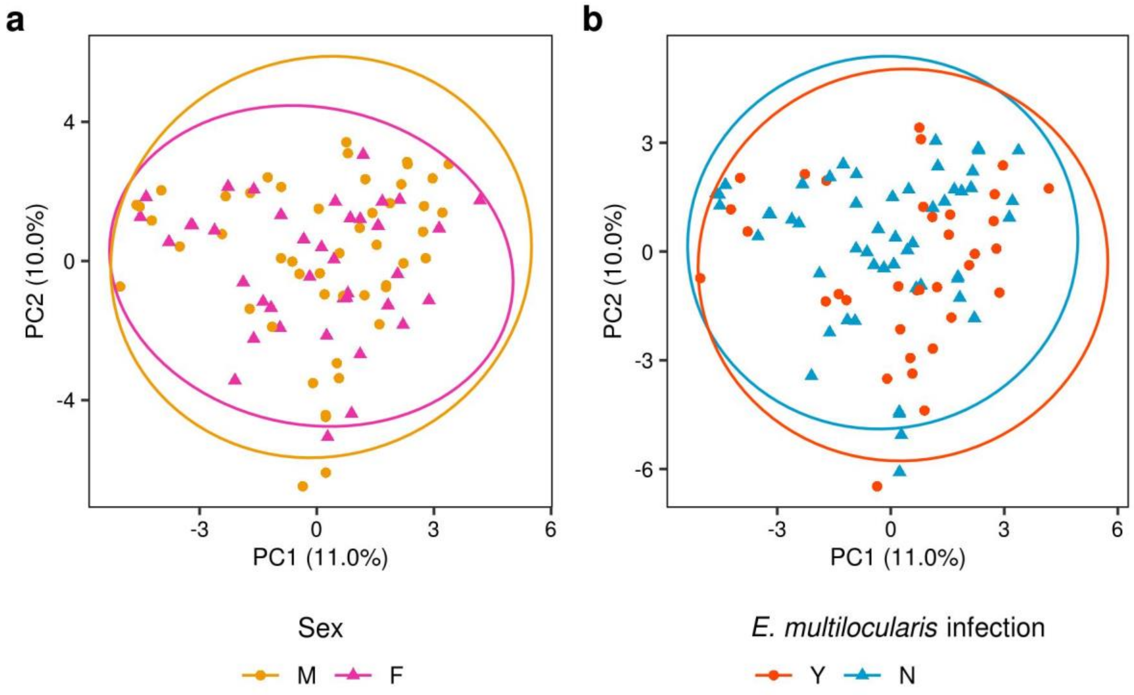

Fig. S9: Effects of sex and *E. multilocularis* infection on microbiome composition.

The Aitchison distance-based ordination from Fig. 2e is shown here colored by (**a**) sex and (**b**) *E. multilocularis* infection status.

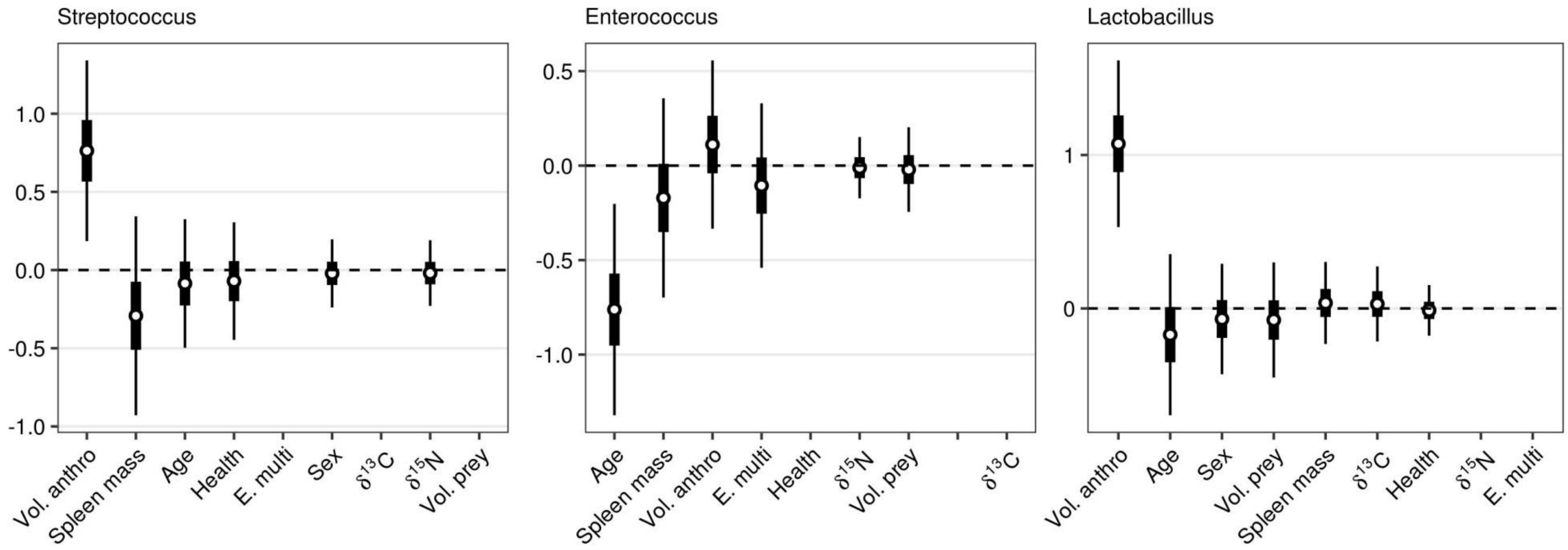

Fig. S10: Generalized linear models for taxa connecting anthropogenic food consumption to health.

The CLR-transformed abundances of genera identified using a regularized canonical correlation analysis (see **Fig. 4**, main text) were used as a response variable in generalized linear models with diet and health measures as predictors. All model subsets were evaluated. Coefficients were averaged across top-ranked models ($\Delta AIC_c < 2$) after being standardized by their partial standard deviation, and predictors were ranked based on the sum of the AIC model weights of the models in which they appear. For each taxon, model-averaged coefficients are shown, ranked along the x-axis in order of decreasing sums of weights.

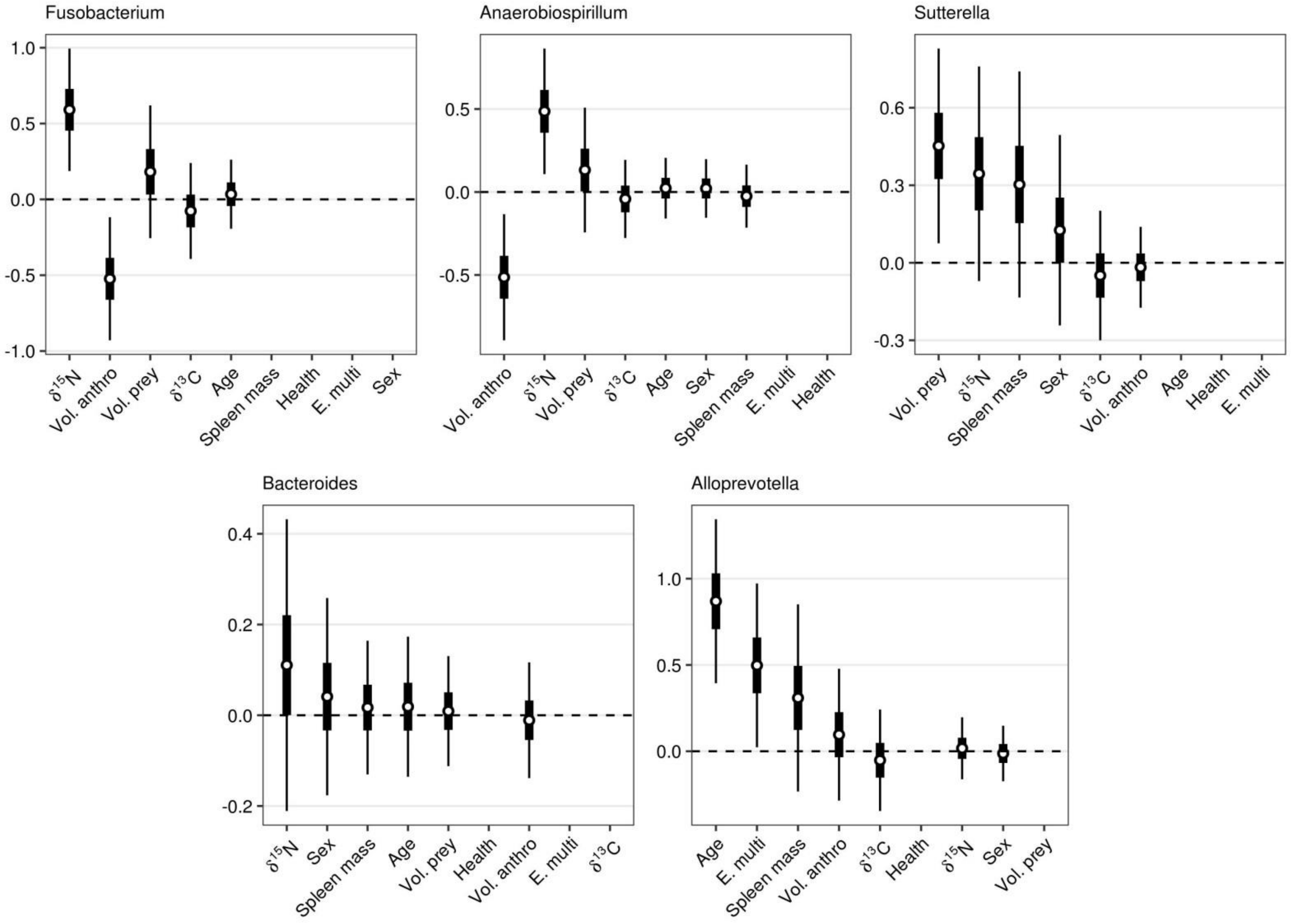

Fig. S11: Generalized linear models for taxa connecting protein consumption to health.

Generalized linear models for genera associated with protein consumption were evaluated in the same way as Fig. S11.

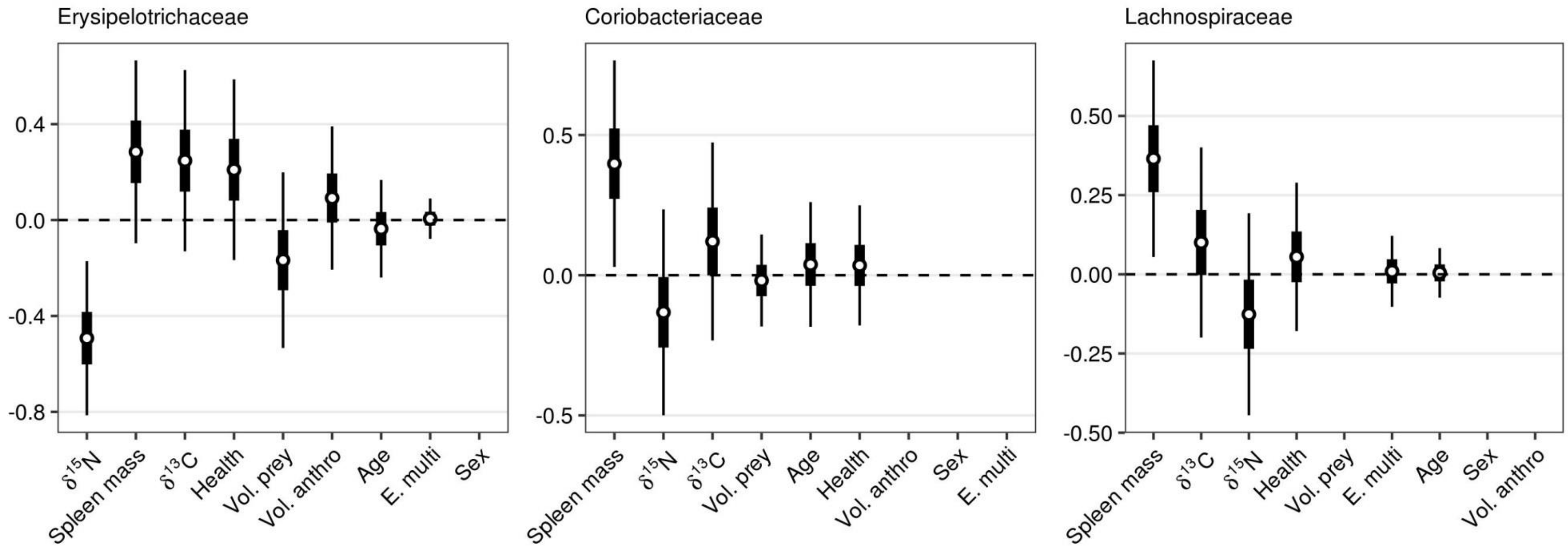

Fig. S12: Generalized linear models for taxa associated with spleen mass.

Generalized linear models for genera associated with spleen mass were evaluated in the same way as Fig. S11.

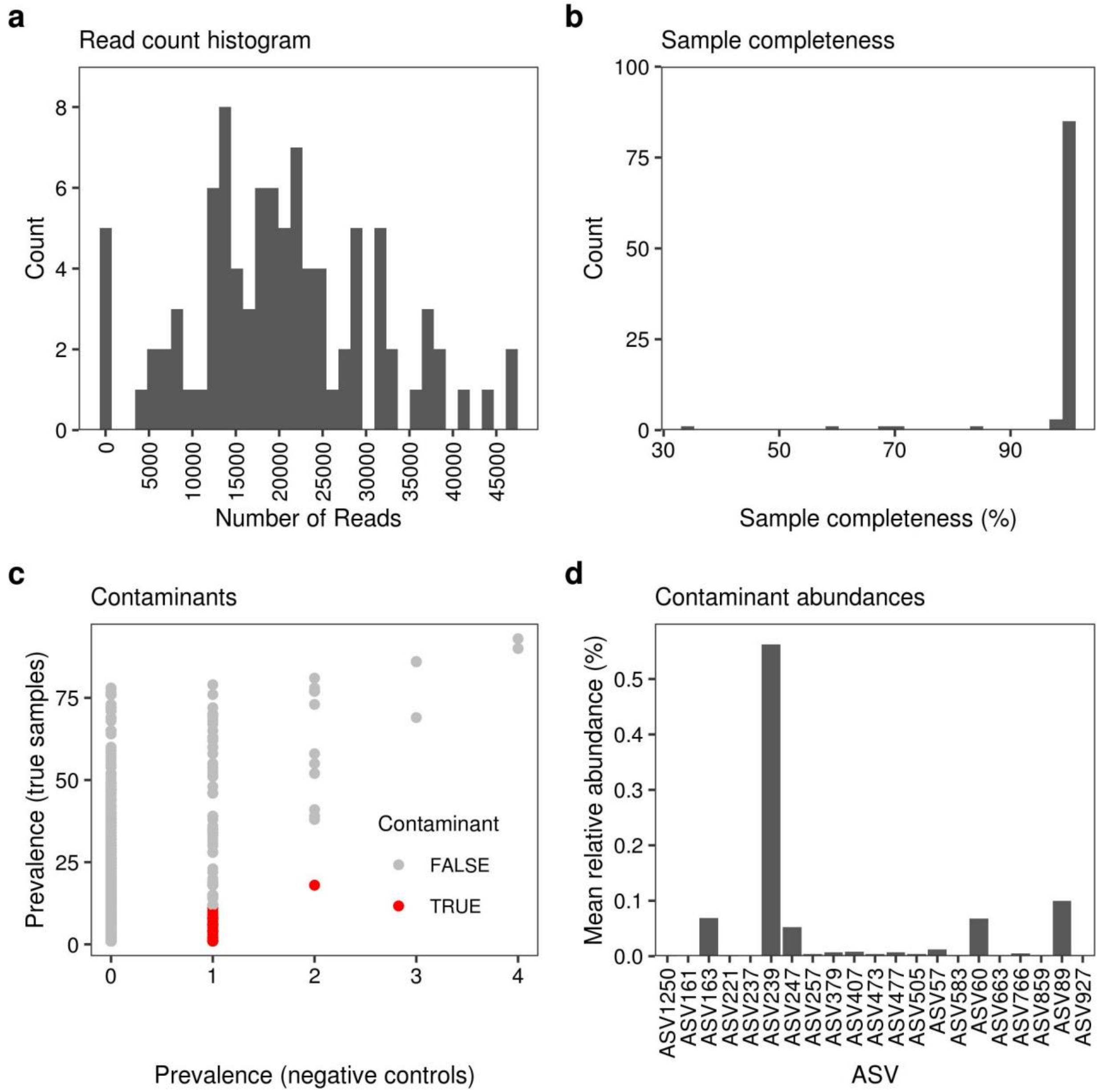

Fig. S13: Sample read count distribution.

(**a**) Histogram of total read counts from each sample, after sequence pre-processing and ASV assignment and prior to any rarefaction or abundance-filtering. The five samples with fewer than 4,500 reads were excluded from all downstream analyses. (**b**) Histogram of sample completeness calculated using *iNext*. Samples with fewer than 4,500 reads had sample completeness values <90%. (**c**) Prevalence of detected contaminants in negative controls and experimental samples, with 21 contaminants removed from future analysis highlighted in red. (**d**) Mean relative abundance of the 21 putative contaminants.

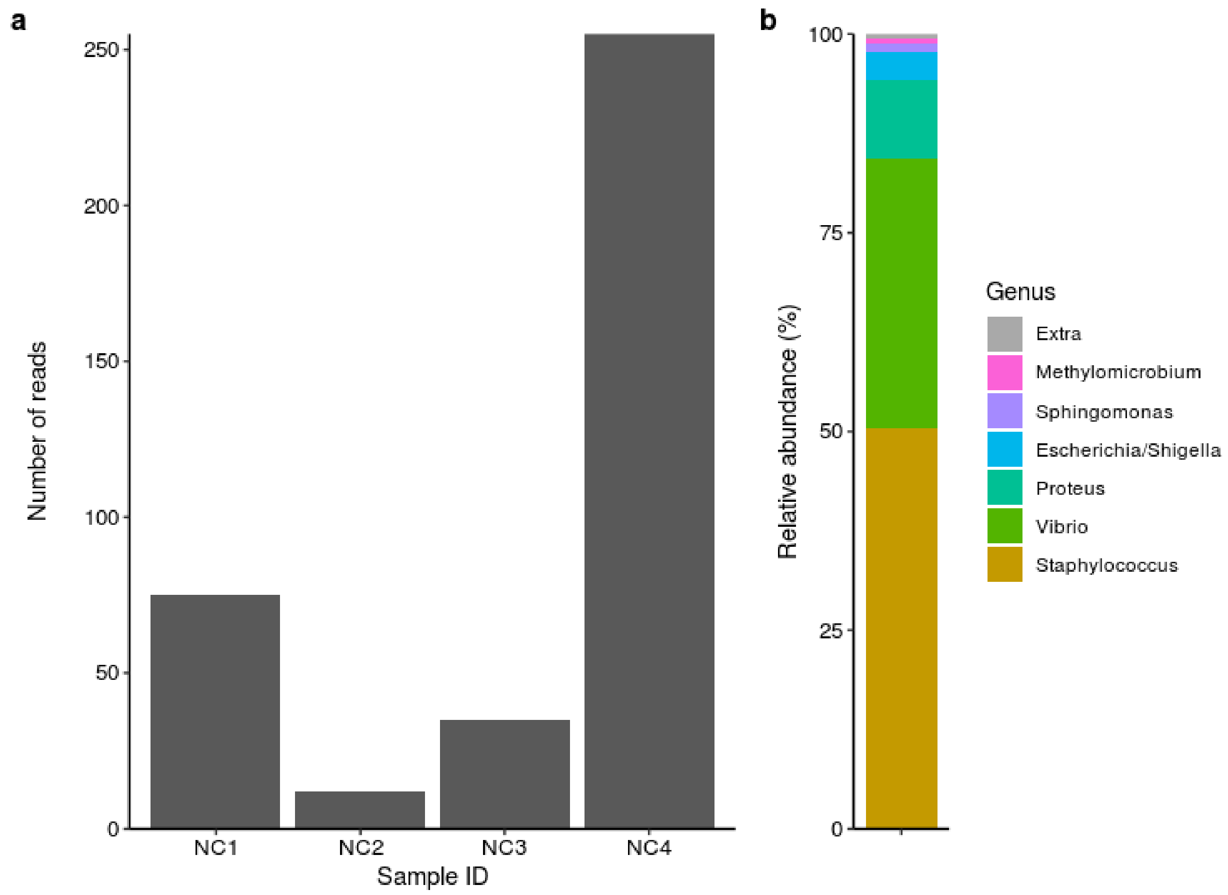

Fig. S14: Sequencing control analysis.

(**a**) Number of reads in each of the four negative control samples. Low read counts suggest minimal contamination, and these negative controls were used as an input to the package *decontam* to remove 22 contaminant ASVs from coyote samples. (**b**) Genus-level mock community relative abundances. We constructed a mock community from DNA available in our lab; the community contained 50% *Staphylococcus aureus*, 30% *Vibrio fluvialis*, 10% *Proteus vulgaris*, 5% *Escherichia coli*, and 2.5% each of *Sphingomonas wittichii* and *Methylomicrobium album* strain BG8. Those abundances were largely preserved in the sequencing results.

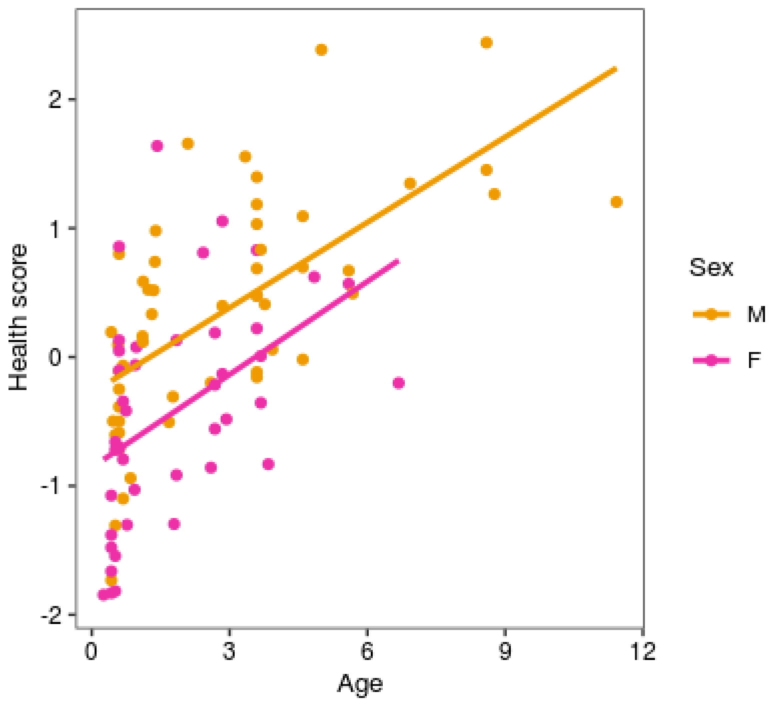

Fig. S15: Collinearity between age and health.

Scatter plot showing best-fit linear regressions between age and health. Due to the strong correlation between age and health, and the limited spread of ages, age was not included as a control in models predicting health. In natural environments, only healthy coyotes live to be old.

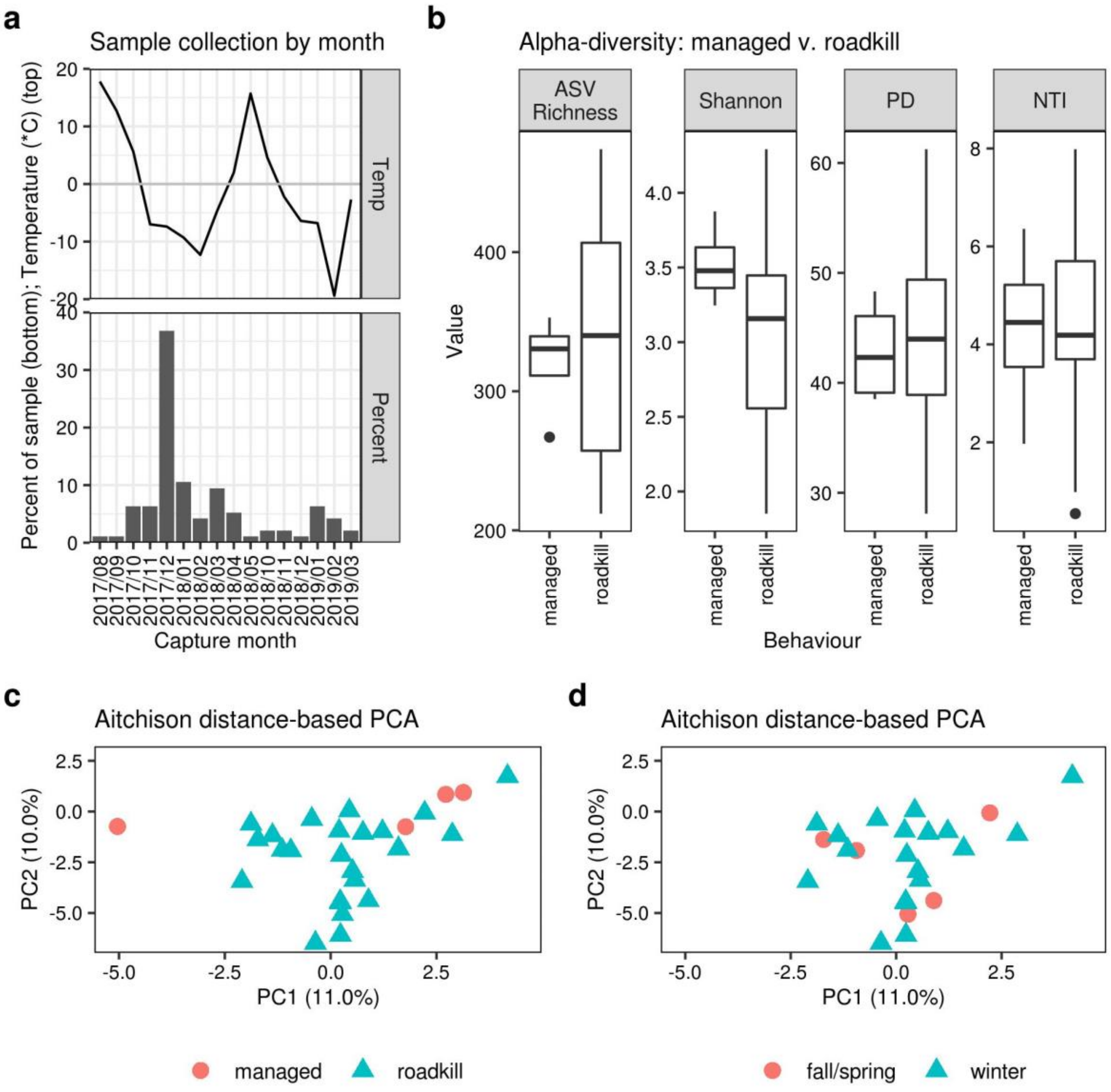

Fig. S16: Sample collection and preservation do not affect microbiome composition. (**a**) Percent of total sample (95 coyotes) collected in the winters of 2017-2018 and 2018-2019, separated by month (*bottom*) in relation to the average monthly temperature (*top*). (**b**) Comparison of alpha-diversity measure for lethally managed coyotes, which were frozen shortly after death, and roadkill coyotes, which were frozen within 24 hours of death. (**c**) Aitchison distance-based principal components analysis (PCA) comparing lethally managed and roadkill coyotes. (**d**) Aitchison distance-based PCA comparing roadkill coyotes captured during the winter (November-April) and those captured in the shoulder season (August-October, May). There are no clear indicators of variability due to the differences in sample preservation associated with sample source or collection method.
